# Estimating geographic subjective well-being from Twitter: A comparison of dictionary and data-driven language methods

**DOI:** 10.1073/pnas.1906364117

**Published:** 2020-04-27

**Authors:** Kokil Jaidka, Salvatore Giorgi, H. Andrew Schwartz, Margaret L. Kern, Lyle H. Ungar, Johannes C. Eichstaedt

**Affiliations:** ^a^Department of Communications and New Media, National University of Singapore, Singapore 117416;; ^b^Centre for Trusted Internet and Community, National University of Singapore, Singapore 117416;; ^c^Department of Computer and Information Science, University of Pennsylvania, Philadelphia, PA 19104;; ^d^Department of Computer Science, Stony Brook University, Stony Brook, NY 11794;; ^e^Melbourne Graduate School of Education, The University of Melbourne, Parkville, VIC 3010, Australia;; ^f^Department of Psychology, Stanford University, Stanford, CA 94305;; ^g^Institute for Human-Centered Artificial Intelligence, Stanford University, Stanford, CA 94305

**Keywords:** Twitter, subjective well-being, language analysis, big data, machine learning

## Abstract

Researchers and policy makers worldwide are interested in measuring the subjective well-being of populations. When users post on social media, they leave behind digital traces that reflect their thoughts and feelings. Aggregation of such digital traces may make it possible to monitor well-being at large scale. However, social media-based methods need to be robust to regional effects if they are to produce reliable estimates. Using a sample of 1.53 billion geotagged English tweets, we provide a systematic evaluation of word-level and data-driven methods for text analysis for generating well-being estimates for 1,208 US counties. We compared Twitter-based county-level estimates with well-being measurements provided by the Gallup-Sharecare Well-Being Index survey through 1.73 million phone surveys. We find that word-level methods (e.g., Linguistic Inquiry and Word Count [LIWC] 2015 and Language Assessment by Mechanical Turk [LabMT]) yielded inconsistent county-level well-being measurements due to regional, cultural, and socioeconomic differences in language use. However, removing as few as three of the most frequent words led to notable improvements in well-being prediction. Data-driven methods provided robust estimates, approximating the Gallup data at up to *r* = 0.64. We show that the findings generalized to county socioeconomic and health outcomes and were robust when poststratifying the samples to be more representative of the general US population. Regional well-being estimation from social media data seems to be robust when supervised data-driven methods are used.

## Significance

Spatial aggregation of Twitter language may make it possible to monitor the subjective well-being of populations on a large scale. Text analysis methods need to yield robust estimates to be dependable. On the one hand, we find that data-driven machine learning-based methods offer accurate and robust measurements of regional well-being across the United States when evaluated against gold-standard Gallup survey measures. On the other hand, we find that standard English word-level methods (such as Linguistic Inquiry and Word Count 2015’s Positive emotion dictionary and Language Assessment by Mechanical Turk) can yield estimates of county well-being inversely correlated with survey estimates, due to regional cultural and socioeconomic differences in language use. Some of the most frequent misleading words can be removed to improve the accuracy of these word-level methods.

Many governments worldwide are incorporating subjective well-being measures as indicators of progress and success (1, 2) to complement traditional objective and economic metrics. Subjective well-being spans cognitive (i.e., life satisfaction), affective (positive and negative emotion), and eudaimonic dimensions (such as a sense of meaning and purpose) (3); most metrics are based on self-report surveys and interviews of individuals, which might be collected annually and aggregated to represent the well-being of regions or nations. Such metrics are time and resource intensive to gather, and there is a growing interest in identifying efficient methods to garner subjective well-being information (4).

Concurrently, social and information exchange has increasingly migrated to digital contexts, including social media platforms. Through language posted online, people leave behind psychological traces that can be mined to address real-world problems. The public nature of Twitter offers a way to augment the theory and practice of psychology and medicine with large-scale data collection. For example, researchers have used Twitter to measure and understand mental illness (5), sleep disorders (6), physical health (7), and heart disease (8).

Studies over the past two decades have established links between autobiographical writing and the psychological well-being of individuals (ref. 9 has a recent review). Twitter-based studies (including those in refs. 10–12) have used different methods to extract overall scores of positive and negative emotion (also referred to as sentiment or valence) through either word-level or data-driven methods (Table 1). Word-level methods, such as the Linguistic Inquiry and Word Count (LIWC) dictionaries (13), involve the use of predetermined or annotated dictionaries (lists of words) that are expected to represent positive and negative emotion and count the relative frequency of words appearing in the dictionary. For example, Golder and Macy (20) applied the LIWC (2007) dictionaries to Twitter posts to track longitudinal variation in affect. Other word-level methods, such as the Language Assessment by Mechanical Turk (LabMT) word list (21) and the Affective Norms of English Words (ANEW) (16), ask raters to annotate words for their valence. For example, LabMT provides the average rater-determined valence (between “sad” and “happy”) for the 10,000 most frequent words in the English language. These crowdsourced ratings have been applied to geotagged Twitter language to estimate the mood of US states and urban and metropolitan statistical areas (10).

**Table 1.** The language-based emotion measures used in this study, which span four main methods: word-level methods and data-driven methods applied at the sentence, user, or county level

Type	Method (source)	No. of features	Categories
Word-level methods			
	LIWC 2015 (13)	1,364	Positive emotion, negative emotion,
			anxiety, anger, sadness
	PERMA dictionary (14, 15)	402	Positive emotion, negative emotion
Word-level annotations	ANEW (16)	1,034	Valence
Word-level annotations	LabMT*^i^* (17)	10,218	Valence
Data-driven methods			
Sentence-level annotations	WWBP affect (18)	7,265	Affect
Sentence-level annotations	Swiss Chocolate (19)	7,168	Positive, neutral and negative emotion
Person-level models	WWBP life satisfaction (this study)	2,000	Cantril Ladder score
Direct prediction Cantril Ladder	County life satisfaction (this study)	2,000	Cantril Ladder score

Data-driven methods involve the use of machine learning to identify associations between the linguistic information contained in the text and its emotional content. The emotional content of sentences or documents (rather than words in isolation) is determined by annotation or based on a self-report survey. Natural language processing methods are used to extract language features, which are then used to predict emotional content using supervised machine learning.

How well do these different methods assess subjective well-being? Previous results with word-level methods are inconsistent (22, 23). At the regional level, LabMT’s state-level happiness estimates show inconsistent associations with life satisfaction reported by the Centers for Disease Control and Prevention (CDC) (10), and at the city level, LabMT’s estimates of happiness were negatively correlated with measures of physical health (24). The unexpected findings may arise from how people use language and differ in their use of social media; alternatively, they could be an artifact of the demographic and geographic effects of aggregating the language of individuals to represent geographies. On the other hand, data-driven methods, which train machine learning models on large corpora and then apply those models to other contexts, have been shown to offer performance improvements over word-based methods for predictive problems (25–27).

In the current study, we compare methods for regional estimates of subjective well-being from social media language against survey-based ground truth measures of county-level evaluative and hedonic well-being (excluding eudaimonic aspects). We use over a billion geolocated tweets from 2009 to 2015 (28), from which we extracted language features, normalized their frequency distributions, and aggregated them to yield county-level language estimates. From these, we extracted emotion/life satisfaction estimates (Table 1).

We aggregated 1.73 million responses to the Gallup-Sharecare Well-Being Index from 2009 to 2015 to obtain county-level measures of life satisfaction, happiness, worry, and sadness. In the primary analysis, we determined the convergent validity between the language-based methods and the Gallup county-level outcomes using an open-source Python codebase (29). We replicated our analyses on county-level health and socioeconomic outcomes to show that the observed patterns generalize beyond self-reported well-being metrics. To account for sample differences, we replicated the primary analysis after poststratifying the Gallup and Twitter samples to match census demographics in age, gender, education, and income. Across a subset of 373 counties, we examined the stability of the findings across time. To investigate the impact of ecological aggregation, we ran parallel analyses across a sample of 2,321 Facebook users. In addition, we conducted a post hoc diagnosis to identify and suggest a solution for the main sources of error in word-level methods.

## Evaluation of Twitter-Based Estimates

Table 2 summarizes the convergent validity from the different methods against the Gallup county estimates. Unexpectedly, among the word-level methods, higher positive emotion/valence estimated from LIWC 2015, ANEW, and LabMT* correlated with lower subjective well-being. For example, both LIWC’s positive emotion dictionary and LabMT correlated negatively (*r* = −0.21 and *r* = −0.27, *P* values < 0.001) with life satisfaction—the most widely used measure of subjective well-being. Similarly, they correlated negatively with happiness and positively with sadness. The PERMA positive emotion dictionary (14, 15, 30) is limited to more unambiguous words and correlated with subjective well-being in the expected direction.^†^ (PERMA is Seligman’s construct of well-being, an acronym for positive emotion, engagement, relationships, meaning, and accomplishment.)

**Table 2.** Pearson correlations (*r*) between Twitter-based emotions and Gallup-Sharecare Well-Being Index estimates across 1,208 US counties



The gray column headers identify the modified LIWC (removed 3 words), LabMT (removed 15 words), andANEW(removed 2 words) dictionaries (in the text). The color indicates the direction and magnitude of correlation; white cells are nonsignificant, and all others are *P* <0.05 corrected for multiple comparisons.

**Table 3.** Pearson correlations (*r*) between Facebook-based emotions and survey responses across 2,321 Facebook users

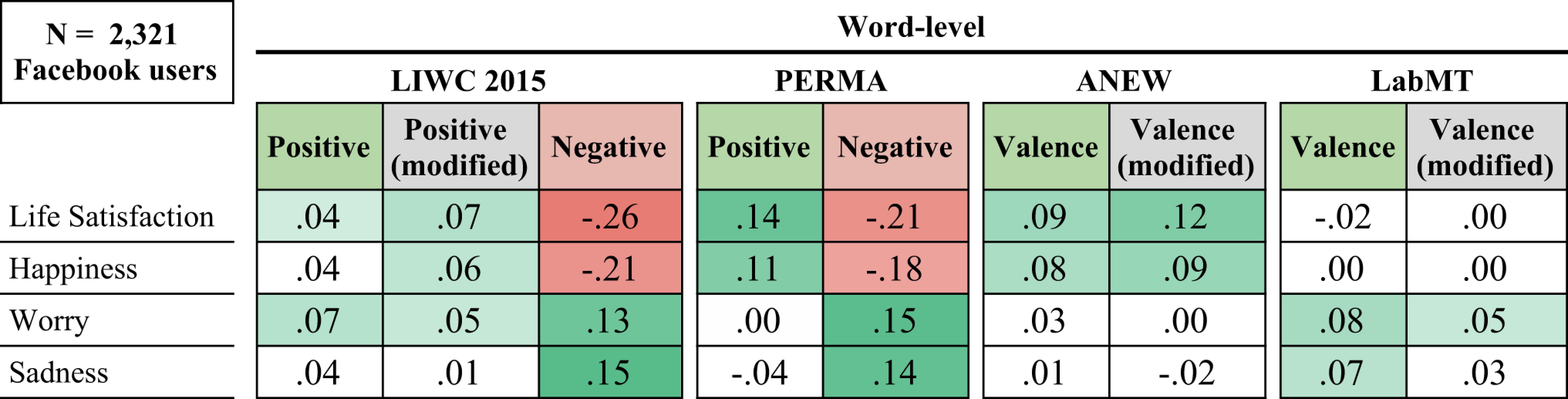

The color indicates direction and magnitude of correlation; white cells are nonsignificant, and all others are *P* < 0.05 corrected for multiple comparisons

The LIWC and PERMA negative emotion dictionaries showed the expected pattern of correlations. Throughout word-level and data-driven methods, negative emotion estimates showed larger and more consistent correlations than their positive counterparts, suggesting that they more consistently captured the absence of well-being on Twitter than its presence. None of the methods predicted worry well, which demonstrated weak correlations across all methods.

In contrast to the word-level methods, the data-driven methods consistently produced estimates that correlated with the Gallup measures in the expected directions, with positive language scores predicting higher life satisfaction and happiness and lower worry and sadness. Data-driven methods thus appear more robust than the word-level methods. Among the data-driven methods, the state-of-the-art sentiment model Swiss Chocolate (19) matched or outperformed the World Well-Being Project (WWBP) affect model (18) and the user-level life satisfaction model that we trained in this study. Direct prediction, also trained by this study, outperformed all other methods (*r* = 0.51 to 0.64, *P* values < 0.001). However, here the models benefited from being directly modeled on Twitter county data and the Gallup outcomes.

### Generalizability to Socioeconomic and Health Outcomes.

To go beyond self-reported measures, we replicated our analyses using county socioeconomic and health variables as dependent variables. We again found that data-driven methods were more robust, outperforming word-level methods.^‡^ For the word-level methods, LIWC’s positive emotion dictionary and LabMT were negatively correlated with an index of socioeconomic status (combining income and education; at *r* = −0.40 and *r* = −0.43, respectively; *P* values < 0.001) as well as positively correlated with CDC-provided measures of poor physical and mental health; therefore, the erroneous associations in Table 2 generalize beyond the well-being outcomes.

### Correcting for Sample Differences.

The population of users in the Gallup and Twitter datasets is notably different from one another and potentially not representative of the US population. Respondents in the Gallup sample were older and wealthier, while those in the Twitter sample were mostly from urban areas and estimated to be younger, with more Hispanics and African Americans than the average US population.^§^ In a supplementary analysis, we poststratified both samples on age, gender, income, and education to render them representative of the county-level US population. For the Twitter sample, we used the language of users to estimate age, gender, income, and education following previously established demographic estimation and selection bias correction methods (31).^¶^ We found that poststratification left the pattern of results largely unchanged; language associations with survey well-being were within *r* = 0.10 of those reported based on the unstratified data.^#^

### Controlling for Demographic and Socioeconomic Confounds.

In order to control for endogenous differences, we added sociodemographic covariates for age, gender, and race when evaluating the language models (*SI Appendix*, Table S10). The resulting pattern of coefficients showed small differences in magnitude when compared with the main results in Table 2. As a stronger test, we entered dummy variables for US states and regions into the regression equations to adjust for unobserved endogenous variables at the state or regional level. Thereby, we only compared counties with counties within the same states and regions. The pattern of correlations was unchanged. Up until this point, these findings suggested that the language-based well-being estimates are not merely attributable to demographic or state-by-state differences in unobserved variables. Finally, when we controlled for income and education, it largely reduced most language associations. This is likely because socioeconomic status was strongly associated with our dependent variable, subjective well-being (e.g., life satisfaction correlated *r* = 0.59 with an income/education index).^‖^ We infer that the variance in the word-level methods overlaps with socioeconomic variance in language use. Some of the data-driven methods captured some variance in Gallup happiness over and above socioeconomic status.

### Stability of Results over Time.

We examined whether our findings were robust to the evolving use of Twitter and well-being trends over time. We repeated our analyses across two shorter windows of time (from 2012 to 2013 and from 2015 to 2016) across a smaller sample of 373 counties for which sufficient Gallup and Twitter data were available. The pattern of results was largely consistent with Table 2. We also evaluated how well models built on 2012 to 2013 Twitter language predicted 2015 to 2016 well-being, finding only a small reduction in performance.**

### Comparison with Individual-Level Language Analyses.

To shed light on the ecological effects of community-level aggregation, we carried out an analogous comparison of language methods at the individual-level across a sample of 2,321 Facebook users who had answered the same survey questions as the Gallup sample. The associations of the LIWC 2015 positive emotion dictionary with well-being were weakly positive (*r* = 0.04, *P* = 0.050), which aligned with previous findings with LIWC 2007 (22). In general, all but LabMT showed weak associations in the expected direction at the individual level. The data-driven methods again produced the expected pattern of correlations, albeit with reduced magnitudes compared with the county level (*r* values < 0.25).^††^

## Word-Level Error Analyses

LIWC’s emotion dictionaries and LabMT are among the most popular tools for assessing emotion through language. To better understand their unexpected pattern of association with county-level well-being, socioeconomic and health variables, we conducted a set of post hoc diagnostic analyses, which suggested that the main sources of error in these word-level methods were due to a few highly frequent words and geographic and cultural variation in language use.

### Word Correlations.

**Fig. 1.**
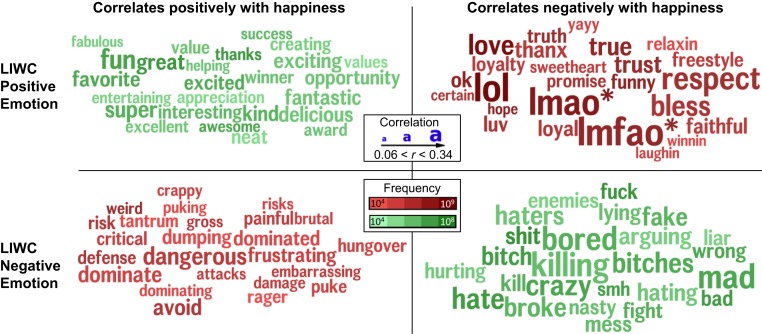
Sources of error in the LIWC positive and negative emotion dictionaries. The matrix illustrates the 25 most frequent words from the two dictionaries that were correlated as expected (green indicates true LIWC positives and true negatives) or opposite to expectation (red indicates false positives and false negatives) with the Gallup happiness item. The size of the word denotes the magnitude of its correlation (0.06 < *r* < 0.34; *P* < 0.05 corrected for multiple comparisons). The shade indicates the normalized frequency, with darker shades reflecting higher frequencies relative to other words.

Fig. 1 depicts a language confusion matrix for the most frequent words in the LIWC positive and negative dictionaries in the form of word clouds. The red diagonal in Fig. 1 identifies correlations that were opposite to expectation. The “false” LIWC positive emotion words in Fig. 1, *Upper Right* provided false signal by correlating negatively with county-level happiness; they were relatively more frequent and more strongly negatively correlated with happiness than the true positive words. They comprise words that may have been synchronously used on social media as markers of flirting, amusement, irony, sarcasm, interjections, and empathy (e.g., “lol,” “lmao,” and “lmfao”) (32). The more the highly frequent word “love” was mentioned, the lower the counties’ well-being [also observed in Eichstaedt et al. (8)] (compare with *SI Appendix*, Table S5). The false LIWC negative emotion words (negative emotion words, which gave false signal because they correlated positively with happiness) (Fig. 1, *Lower Left*) were of higher complexity (e.g., “dangerous,” “frustrating,” “embarrassing,” “critical,” and “weird”) and were likely used by older populations with relatively higher education (33). Similar patterns were observed for LabMT.^‡‡^

### Highly Frequent Words.

The frequency distribution of words in the English language is Zipfian (follows a power law distribution): relatively few words account for a near majority of occurrences. The same is true for words in a dictionary. Specifically, the words lol, love, and “good” were the most frequent words in the LIWC positive emotion dictionary, accounting for about 25% of the county word occurrences. Similarly, these words and some pronouns (including “you,” “my,” and “me”) accounted for roughly 20% of the (weighted) positive valence measured by LabMT.^§§^ We found these few highly frequent words to have negative correlations with both well-being and income (*SI Appendix*, Fig. S3). Removing them uniformly improved convergence with Gallup measures (gray columns in Table 2). For example, the modifications improved LIWC’s prediction of happiness from *r* = −0.13 to 0.13 and LabMT’s from *r* = −0.07 to 0.16.^¶¶^

### Mapping False Positive Emotion Words.

**Fig. 2.**
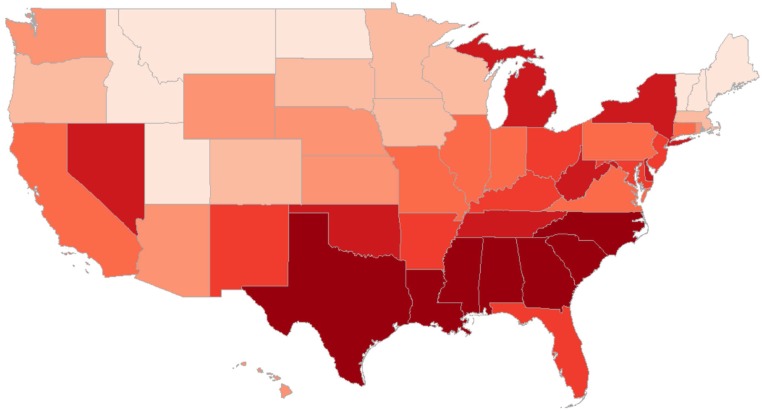
The relative frequency of false LIWC positive emotion words across the United States. States with a darker shade of red had relatively higher numbers of positive emotion words that correlated negatively with county Gallup happiness (Fig. 1, *Upper Right*) at *P* < 0.05, controlling for multiple comparisons.

Fig. 2 illustrates the relative frequency of false LIWC positive emotion words (as in Fig 1, they were the positive emotion words that falsely had a negative correlation with Gallup happiness). The map suggests a geocultural divide: false LIWC positive emotion words were used more frequently in the South and the Southeast, which roughly corresponds with the Mason–Dixon Line.^##^ We infer that our Twitter-based LIWC positive emotion measurements captured how different regions of the United States use these words differently. Furthermore, these usage differences overlapped with the socioeconomic gradients across the United States in ways that produced the unexpected negative correlations with well-being. Controlling for income and education reduced some of the unexpected associations of these words with well-being—and of the overall LIWC dictionary—to insignificance.***

### Context Effects.

The LIWC positive emotion dictionary captures a heterogeneity of language use. To better understand it, we considered how many of the words contained in the LIWC positive emotion dictionary are also included in other LIWC dictionaries capturing different concepts (the overlapping dictionary words accounted for 1.1% [religion] to 26.6% [netspeak] of positive emotion word occurrences) (Table 4 and *SI Appendix*, Table S15).

**Table 4.** Pearson correlations (*r*) between Gallup-Sharecare Well-Being Index-based estimates and Twitter use of subsets of LIWC positive emotion words that co-occur with other LIWC dictionaries across 1,208 US counties

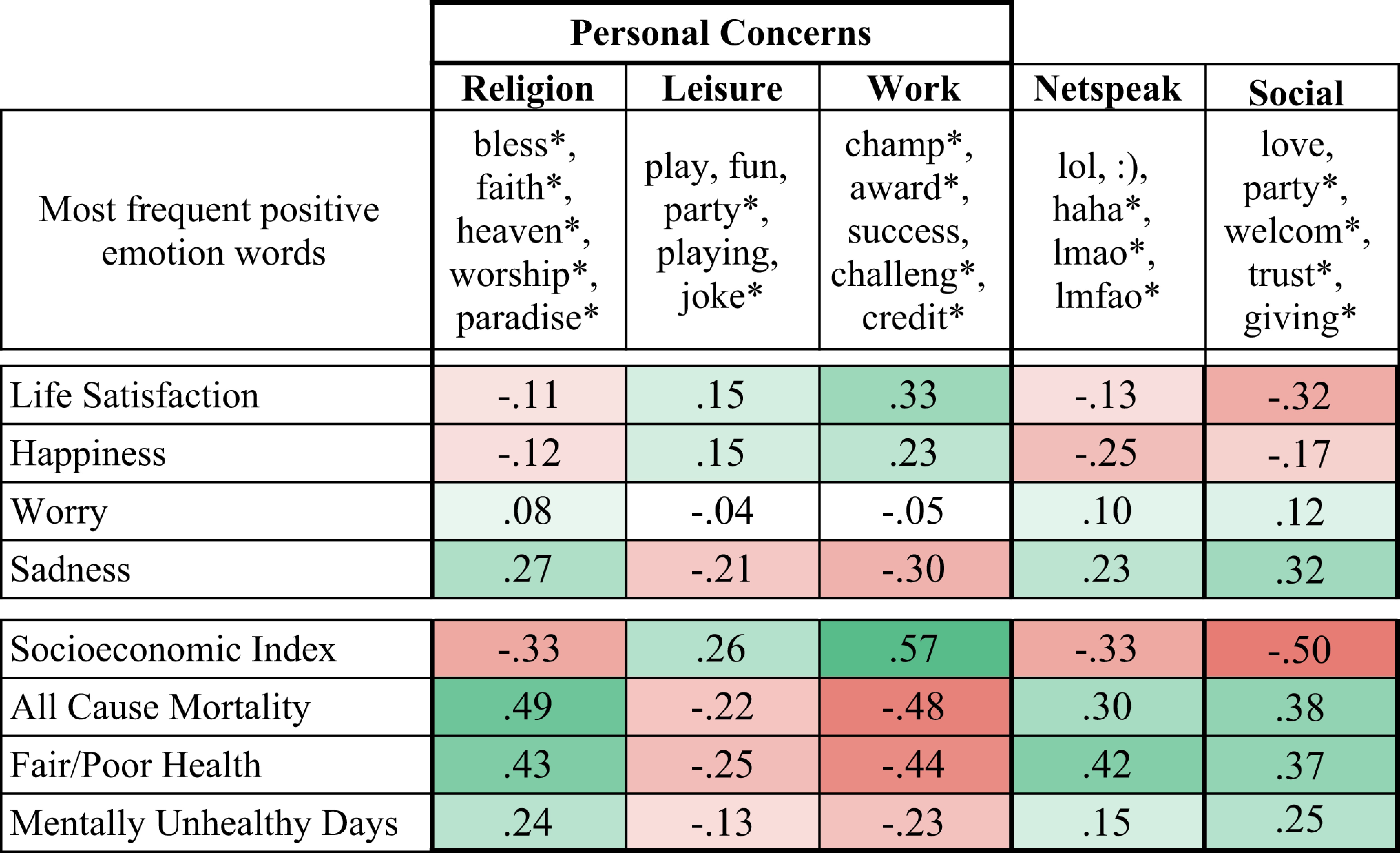

Color indicates direction and magnitude of correlation; white cells are nonsignificant, and all others are *P* < 0.05 corrected for multiple comparisons

This demonstrates that even a dictionary intended to measure a single construct (such as positive emotion or valence) may inadvertently aggregate over different types of language use and speech acts—which themselves may differ substantially in their geographic association with well-being and income. In the context of Fig. 2, we can infer that language related to “work” and professions was indicative of higher income in the North (34), thus explaining correlations of *r* = 0.33 (*P* < 0.001) with county-level life satisfaction and *r* = 0.57 (*P* < 0.001) with socioeconomic status (income and education).

## Discussion

The psychological signal left behind in digital traces on social media makes it possible to unobtrusively monitor the well-being of regions (US counties in this case). Language analysis is the most widespread method to derive emotion or well-being estimates from such data. This study demonstrates that Twitter language can be used to measure the well-being of large populations if robust data-driven methods are used, which seem to circumvent errors associated with word-level methods. We found that data-driven well-being estimates also predicted US county economic and health outcomes. They were largely unchanged when correcting for sample biases through poststratification, when including demographic covariates, or when comparing only counties to counties within states. We found that the pattern of correlations with county Gallup estimates was stable over time. Regarding the choice of language analysis method, our study had three main findings.

First, word-level methods for subjective well-being measurement should be used with caution. One of the primary difficulties in estimating psychological states for geographies using social media arises from applying methods designed to measure the emotion of sentences of individuals to the language of regional populations. The language of regions differs culturally, such as the South using more religious language. When these cultural differences interact with socioeconomic gradients, these differences may invert the expected relationship between word-level estimates and well-being and health outcomes.

Second, most of the discrepancies observed for word-level methods seem to be driven by the use of a few frequent words (such as lol, love, and good). Stylistic markers such as lol can be used to convey a variety of emotions (32); they may also symbolize meanings that are specific to cultures and communities. Removing these words from LIWC, ANEW, and LabMT dictionaries reduced the negative associations with Gallup happiness and thus, improved the convergence with survey-reported county-level well-being.

Third, data-driven language models using supervised machine learning based on the sentence-, person-, or county-level training data seem to generate valid geographical estimates of well-being. The same language models worked consistently across counties and individuals. Methods that directly predict county well-being from county language seemed able to capture counties’ social and socioeconomic context and explain the regional variance in well-being over and above socioeconomic indicators.^†††^ These models offer opportunities to augment other methods of spatial estimation by providing estimates with higher temporal resolution than annual surveys and by providing estimates for regions that are insufficiently covered by other sampling methods.

Our study also had three main findings about what explains the difference in performance between word-level and data-driven county-level well-being estimation. First, cultural norms may shape the associations between world-level estimates, well-being, and health. To the extent that social media users underreport socially undesirable and overreport socially desirable emotions, methods that rely only on emotion language may misestimate well-being. These estimation errors may be critical to study subpopulations that share different cultural notions of ideal affect, such as Asian Americans’ preference for low-arousal emotions (35)—as a result, emotion-focused language estimates may underestimate their well-being. In contrast, the use of the full vocabulary considers other kinds of signals, such as function words (e.g., “of,” “the,” “for”), which can also represent higher cognitive processing that covaries with subjective well-being (36). In support of this claim, employing 73 LIWC dictionaries as features in direct county-level prediction yielded a performance nearly at par with the data-driven Twitter language model.

Second, the data-driven methods do not inherit the annotator biases of word-level methods (as used by ANEW or LabMT), which may lead to words such as “conservative” and “exams” acquiring a negative valence and “baby” acquiring a positive one. Such annotations may reflect the view of the annotators of these words outside the broader cultural and socioeconomic context of these words and may differ by the cultural context of the annotators. Sentence- and person-level methods incorporate broader semantic contexts beyond single words.

Third, data-driven methods can capture the socioeconomic variance present in the samples on which they were trained. At times, these language associations deviate from the apparent valence of words outside their socioeconomic context. For example, individuals with higher socioeconomic status and well-being more frequently mention “taxes” and “penalty”—while negatively valenced for individuals, these are markers of relative prosperity at the county level. Similarly, “mortgages” are indicative of homeownership and socioeconomic status (37). Data-driven models capture these words as markers of higher well-being despite their apparent negative valence.

This study focused on language measures of valence and emotion as estimates of county well-being. Care is needed when pursuing the reverse analytic strategy and interpreting language correlations to characterize the well-being of individuals. For instance, many studies have shown that stronger religiosity (38, 39) and sociality (40, 41) benefit well-being. However, correlations with religious language or social words such as love may suggest the opposite at the population level unless socioeconomic contexts are properly considered.

### Limitations.

Limited by the availability of county-level Gallup data, we evaluated Twitter methods against county evaluative and affective dimensions of subjective well-being but did not include eudaimonic measures capturing meaning and purpose (42). Associations between eudaimonic measures and language-based estimates may differ.

While Twitter provides an unprecedented opportunity to observe the natural communications in communities, only a small fraction of Twitter posts has geolocation information (28). Still, the sample size of users who can be geolocated (5.73 million in this study) matches or exceeds the largest phone-based survey efforts. Our analysis was limited to English language posts on Twitter and thus, may have missed signals from other languages prominently used in the United States, such as Spanish and Chinese. Twitter’s user base is not representative of the US population, and many people do not use Twitter—concerns that we addressed 1) through testing the Twitter language models against the Gallup samples using random dialing and 2) through replicating our analysis on samples that were poststratified toward age, gender, income, and education distributions reported by official sources. It is not clear that regular social media users are substantially different from nonregular users; for example, recent work in a large cohort study of females aged 53 to 70 found a very similar profile of sociodemographic and psychosocial factors across both groups (43).

The findings reported in this paper are correlational and do not intend to make causal claims. They provide a snapshot of community health and well-being correlates, but as internet language evolves (32, 44, 45), the correlations between social media language features and well-being are likely to change over time. Although the data-driven methods in this paper, such as the WWBP affect model and the WWBP life satisfaction model, were trained on Facebook posts and then applied to Twitter, we do not expect this to have substantially affected their performance when applied to the county level (46, 47).^‡‡‡^

## Materials and Methods

Full methods are in *SI Appendix*.

### County Twitter Data.

We used the County Tweet Lexical Bank from ref. 28, which comprises language estimates of US counties and corresponds in time to the Gallup well-being dataset.^§§§^

### Gallup-Sharecare Well-Being Index.

We included 1,208 counties that had at least 300 Gallup respondents and sufficient Twitter language. To facilitate secondary poststratification analyses, we limited the sample to respondents for whom age, gender, income, and education were available before aggregating the well-being estimates to the county-level, which reduced the sample by 1.6%. In total, we aggregated 1,727,158 Gallup survey responses.^¶¶¶^

### Individual-Level Data.

We recruited adults in the United States via Qualtrics for a well-being survey, which included the same well-being items as used by Gallup; 2,321 individuals consented to share their Facebook data and had posted at least 100 posts on Facebook. Emotion measurements based on word-level and data-driven methods were obtained and compared against self-reported well-being. This study was approved by the Institutional Review Board at the University of Pennsylvania.^###^

## Data Availability

The Gallup-Sharecare Well-Being Index data are available by institutional subscription. County language estimates are available in the WWBP GitHub repository (https://github.com/wwbp/county_tweet_lexical_bank) (48). Replication code and the WWBP life satisfaction model are contained in the Open Science Framework archive (https://osf.io/jqk6f/) (49).

## Supplementary Material

Supplementary File

## Footnotes

*Following ref. 17, we removed “neutral” words with 4 < valence < 6, leaving 3,731 words.

^†^*SI Appendix*, Table S16 has details on the approaches, and *SI Appendix*, Table S3 has extended results covering additional word- and sentence-level methods.

^‡^*SI Appendix*, Table S5 has the detailed results.

^§^*SI Appendix*, Table S6 has a general overview of the response biases.

^¶^Details on the model accuracies are in *SI Appendix*, Table S7.

^#^The poststratification process is validated in *SI Appendix*, Table S8.

^‖^*SI Appendix*, Table S18 has details.

**Additional information is in *SI Appendix*, Table S11.

^††^*SI Appendix*, Table S13 has the full results.

^‡‡^More details are in *SI Appendix*, Fig. S3*B* and the discussion of *SI Appendix*, Fig. S4.

^§§^Here, we consider words with a LabMT valence more than six as positive following ref. 17.

^¶¶^*SI Appendix*, *SI Text*, Fig. S3, and Table S14 has more details.

^##^The border between the Civil War North and South.

***Additional information is in *SI Appendix*, Fig. S3*B* and Table S10.

^†††^Additional information is in *SI Appendix*, Table S3*C*.

^‡‡‡^Additional information is in *SI Appendix*, *Supervised Person-Level Methods* and Table S2.

^§§§^*SI Appendix* and ref. 28 have further details on the language data extraction process.

^¶¶¶^*SI Appendix*, Fig. S1 shows the inclusion criteria.

^###^Dataset statistics are provided in *SI Appendix*, Tables S1*A* and S12.
