# Supplementary material for: Estimating geographic subjective well-being from Twitter: A comparison of dictionary and data-driven language methods
Source: Proc Natl Acad Sci U S A. 2020 Apr 27;117(19):10165–71. doi: 10.1073/pnas.1906364117 (PMC7229753; doi:10.1073/pnas.1906364117)
Supplement: Supplementary File [file pnas.1906364117.sapp.pdf]

PNAS
www.pnas.org

Supplementary Information for

Estimating geographic subjective well-being from Twitter: a comparison of dictionary and data-driven language methods

Kokil Jaidka, Salvatore Giorgi, H. Andrew Schwartz, Margaret L. Kern, Lyle Ungar and Johannes C. Eichstaedt

Kokil Jaidka, Johannes C. Eichstaedt.
E-mail: jaidka@nus.edu.sg, johannes.stanford@gmail.com

This PDF file includes:

Supplementary text
Figs. S1 to S4
Tables S1 to S20
References for SI reference citations

www.pnas.org/cgi/doi/10.1073/pnas.1906364117

Supporting Information Text

Across all the Figures and Tables in this study, significant correlations have been denoted by using a red/green shaded cell, where the gradient of the red/green shade denotes the strength of the negative/positive correlation. Significance was calculated row-wise, after Benjamini-Hochberg correction ($p \leq .05$) was applied over all the language features and covariates reported in any given row.

Dataset statistics

Figure S1 details sample composition and drop-out for US counties. Only counties with 300 or more responses during the 2009-2015 time period were selected. Responses with incomplete demographic information were filtered out (to allow for post-stratification based on age, gender, income, and education), resulting in fewer than 300 responses per county in some cases (a total loss of 1.6% of participants). Of 3,142 US counties, 1,208 counties had sufficient Twitter and Gallup data and were included in the study. Table S1 describes the average, median, and range of respondents per county. The Table also provides the survey items that were used to measure well-being within the Gallup Sharecare well-being Index (1), and indicates the response scale, mean and standard deviation per item.

Description of the language-based methods

Word-level methods measure emotion or well-being by counting the relative frequency of usage of words arranged in different categories. For example, the Linguistic Inquiry and Word Count (LIWC) 2015 (2) or the PERMA dictionaries (3, 4) comprise words organized to capture linguistic, psychological, or cognitive constructs. The list of words and valence, arousal, and dominance scores in the Affective Norms of English Words (ANEW) (5), Warriner's extended ANEW (6), and Language Assessment by Mechanical Turk (LabMT) (7) were created based on an average or agreement among multiple annotators, who labeled individual words for their emotional content.

In sentence-level methods, supervised machine learning models infer word-level emotion or well-being ratings from corpora of annotated sentences. The WWBP Affect lexicon (2), National Research Council's Hashtag Emotion lexicon (8) and Swiss Chocolate (9) are examples of sentence-level methods for measuring emotion.

In person-level methods, survey respondents who self-report their Life satisfaction scores also share their social media posts, thus producing a language corpus labeled with the survey-based Life satisfaction of their authors. Supervised language models are then trained on the linguistic features extracted from the corpus to identify a list of significant features and corresponding weights. We evaluate such a model, trained on the social media language associated with self-reported responses to the Cantril Ladder Life Satisfaction question in an online survey administered through Qualtrics to a random sample of 2,143 Facebook users out of a larger sample of 2,321 users; the data collected are described in a subsequent section. The data for $N = 178$ users were held out for validation purposes. A few previous studies (10, 11) have explored the use of person-level models of personality and stress trained on individuals' social media posts for predicting their regional variations across the United States; however, to our knowledge, no similar study has been conducted for estimating regional well-being.

Details on the extracting emotion measures from language.

Word-level methods. We calculated the county estimates by adding the word counts of words contained in the respective dictionaries. Wherever methods provided valence or affect scores, we used them as weights. The LabMT valence dictionary contained continuous valence scores for words derived from annotators (ranging from 1 to 9), which we mean-centered.

Supervised sentence-level methods. Pre-trained classifiers and language models trained on labeled social media posts can predict the emotion labels of text as a function of its words. In the case of the WWBP Affect model (12), linguistic features at the post level are typically sparser than at other aggregation levels (e.g., a single post vs. the user's entire timeline of posts); accordingly, word features were binary-encoded (1 if the word was present in the post, 0 if not). We took the total number of times a user mentioned a specific word and normalized it by their post count. Finally, user-level features were averaged to the county level.

Supervised Person-level methods. Individuals' social media language were labeled according to their self-reported Life satisfaction measured by Cantril's Ladder. This method is thus more directly targeted at measuring evaluative well-being, as compared to the emotion-focused word- and sentence-level methods. We extracted 2,000 topics from the social media posts of the Facebook users in our sample. The topic features were available from previous work (13, 14) and were derived via Latent Dirichlet Allocation (LDA) (15) over a large Facebook corpus. We then trained a language model comprising these topics against the survey-reported Life satisfaction scores of 2,143 Facebook users (described in further detail below). We call this the WWBP Life satisfaction model and subsequently used this model to predict the well-being for each county in our Twitter sample.

Some words and discourse features, such as 'RT' and '#,' are more likely to occur on Twitter than on Facebook. Differences also arise when different social media platforms differ in their technological and social affordances, leading to differences in emotional expression (16), and the quality of communication (17). Consequently, the predictive performance of language models may change when they are applied to predict user traits from the language of a different domain. Some studies at the user-level have reported that there is a marginal *improvement* in predicting user traits when the validation data comprises Twitter posts if the pre-trained language models are trained on Facebook rather than Twitter posts (16, 18). In contrast, other studies have reported a drop in performance of 2-10% when models trained on Facebook were applied to Twitter to predict

users' age and gender, and vice-versa (19). At the regional level, previous work has reported only a small effect of normalizing word frequency distributions on the ultimate predictive performance (10). Given the mixed findings, in the present scenario, we evaluated the effect of cross-platform prediction by training two language models on smaller subsets of 522 users from our Qualtrics panel (described in detail below), for which their Twitter posts were also available, and their posts included at least 500 words each on Facebook and Twitter. For these 522 users, we trained two language models against their survey-reported Life Satisfaction, once taking only their Facebook posts and then taking only their Twitter posts. Then, we applied these models to predict Gallup Life Satisfaction at the county level, as before. Table S2 reports the correlations between the Gallup well-being outcomes and the values predicted by the two comparable language models. The resulting pattern of correlations is very similar (e.g., the person-level model trained on Facebook language and applied to county Twitter data reaches r = .38 correlation with county Gallup Life Satisfaction, compared to r = .33 for the person-level Twitter model). Thus, we observed no performance degradation when applying Facebook models to Twitter as compared to applying Twitter models to Twitter language.

Direct prediction. We trained a predictive model to directly predict county-level well-being by training a language model comprising the language features representing each county's Tweets. All predictions were made for counties other than the ones used to train the model in a 10-fold cross-validation framework.

Experimental setup for direct prediction methods. We used a 10-fold cross-validation framework: over ten iterations, we trained ten language s on 90% of the counties and evaluated the accuracy of its predictions on a held-out 10% of the counties. Finally, we report the predictive performance as Pearson's correlations between all the predictions on held-out counties, and the county-level Gallup well-being scores. To reduce the high dimensionality of the language feature space, we used a combination of feature selection, principal components analysis, and ridge regression to avoid overfitting models. We first removed all features whose distributions did not correlate with the outcome at a family-wise error rate alpha of 0.60 and then conducted randomized principal component analyses to reduce the dimensionality of the features. The resultant principal components were used as predictors in ridge regressions.

As shown in Table S3c, four models were evaluated using a combination of socioeconomic, LIWC-based features, words, and LDA topics (statistically derived sets of words that tend to co-occur (15)); as the independent variables in separate models and considered Life satisfaction as the dependent variable. Model 1 (SES) only included the socioeconomic index [i]. Model 2 (All LIWC dictionaries) used all the 73 LIWC dictionaries with ridge regression. Model 3 (All language) used all the LDA topics with ridge regression, with the feature reduction steps. Model 4 (All language + SES) combined the socioeconomic variables with the predictors of model 3. In model 4, we first trained ordinary least square models on the socioeconomic index. Next, we trained the ridge regression model by using Twitter language to predict the residuals of the first model, to distinguish the contribution of the single socioeconomic index from the many Twitter features, and to identify the actual contribution of the Twitter language over and above socioeconomic factors. The predictions on held-out data were evaluated as Pearson's correlations with the four Gallup county outcomes (Life satisfaction, Happiness, Worry, and Sadness).

Predictive Performance Evaluation. The predictive performance of all four model classes was evaluated as Pearson's correlations between the predicted well-being and the actual Gallup well-being outcomes. In the case of the person-level and county-level language models, Pearson's correlations were calculated between the model predictions and survey-reported outcomes over the ten folds of held-out data. The significance of the differences between the models' performance was assessed using a paired t-test over the magnitude of the models' residuals. We anticipated that Life satisfaction and Happiness would be positively associated with the use of positive emotion words in social media posts. In contrast, Sadness and Worry would be negatively associated with the use of positive emotion words and positively associated with negative emotion words.

Predictive Performance Detailed Results. Table S3 provides detailed results in terms of the predictive performance of Twitter-based well-being measurements, measured as Pearson's correlation against the Gallup poll results across 1,208 counties. The choice of language analysis technique can play an important role in the accuracy in measuring psychological constructs.

Table S3a extends the results presented in Table 2 and includes the predictions based on the extended Warriner's lexicon. Among the methods using positive emotion, the PERMA Positive emotion measure was the best estimator of emotion at predicting Life satisfaction. In Table S3b, the Anticipation sentence-level model from the NRC Hashtag Emotion lexicon demonstrated the best performance at predicting Life Satisfaction ($r = .38$, $p < .001$).

Table S3c shows the results from direct prediction – a Pearson's correlation over the ten (held out) folds for models trained on all of the LIWC features, and the entire county-level vocabulary in the 2000 topics pre-trained on a social media corpus by previous work (13). Language models trained on the 2000 LDA topics predicted well-being at $r = .51$ to $.64$ ($p < .001$). Twitter county language significantly improved upon SES-based predictions for Happiness, Worry, and Sadness. The Table also shows that using all the LIWC categories in a direct prediction method led to a model with performance comparable to the model based on all Twitter language modeled as LDA topics ($r = .46$ to $.58$, $p < .001$).

Generalizability to other county-level socioeconomic and health outcomes

As a test of robustness, we tested the extent to which data-driven methods outperformed other methods for predicting other county-level demographic outcomes and health factors.

[i] See Table S4 for details on socioeconomic index

Health Data. The Behavioral Risk Factor Surveillance System (BRFSS) is a population-based cross-sectional telephone and cell phone health survey of adults in the US aged over 18 years. We obtained the following health factors corresponding to health and mortality. Information about these outcomes and transformations applied are provided in Table S4:

- % fair or poor health
- All-cause mortality
- Mentally unhealthy days

Census data obtained from the 2015 American Community Survey's five-year estimates (20) were used as controls.

Socioeconomic index. Studies have identified strong associations between socioeconomic status and Life satisfaction (21, 22) but generally weaker or curvilinear associations between socioeconomic status with affective well-being (23). We created a socioeconomic index as a baseline to understand the predictive power of county socioeconomics and the relative accuracy provided by Twitter measurements over and above this baseline. We obtained the county-level socioeconomic factors from the American Community Survey's five-year estimates, with the percentage of the population with a bachelor's degree or higher and the median per capita income. Because income and education are highly correlated, we created a composite county-level socioeconomic index by first standardizing and then averaging these measures (analogous to (24)).

Generalizability Supplemental Results. Table S5 shows the correlations between Twitter-based emotion measurements and county-level health outcomes. We observed correlation patterns very similar to the previous results: estimates from supervised language models at the post-level, person-level, and county-level showed the strongest correlations with a variety of demographic and health outcomes. As seen in our prior results, against expectation, counties with higher LIWC positive emotion scores or higher LabMT scores were more likely to suffer from poor health ($r = .37$ and $r = .25$, $p < .001$) and higher mortality ($r = .26$ and $r = .32$, $p < .001$). Annotation-based models, such as the WWBP Affect and the Swiss Chocolate models, reported moderate associations with county demographics and health outcomes in the expected directions. Higher positive emotion correlating with higher socioeconomic status ($r = .39$ and $r = .40$ respectively, $p < .001$) and better health ($r = -.26$ and $r = -.33$ against fair/poor health, $p < .001$). Finally, the direct prediction models offered a greater improvement over other methods as compared to the prediction of well-being ($|r| > .51$, $p < .001$). As the general pattern of findings replicates to other socioeconomic and health variables at the county level, we conclude that our main takeaways are not contingent on the specific choice of Gallup well-being outcomes.

Correcting for Sample Differences

Even with 1.73 million responses over eight years, Gallup's daily surveys offer insufficient data for estimation of county-level well- being in most US counties. Figure S2 shows the Life satisfaction scores for the 1,208 US counties (of 3,142 total counties) for which at least 300 responses were available between 2009 and 2015. A skew in the coverage created a convenience sample with certain demographic biases.

When missing or non-representative data is correlated with a target outcome, then excluding observations can lead to false inferences (25). Non-responses create systematic biases in the sample when they are correlated with differences in well-being. We tested for a non-response bias by correlating the presence or absence of counties in our Gallup and Twitter datasets. Table S6 shows the likelihood of a county with a higher percentage of a demographic attribute of being present in the initial Gallup, the initial Twitter dataset, and the final, filtered dataset of 1208 counties used in the current paper. The negative correlation with % rural population ($r = -.61$ among Gallup counties; $r = -.60$ among Twitter counties, $p < .001$) implies that both Gallup and Twitter were likely to under-represent counties with a larger rural population. The negative correlation with % male population ($r = -.20$ among Gallup counties; $r = -.22$ among Twitter counties, $p < .001$) implies that both Gallup and Twitter were likely to under-represent counties with a larger male population. Gallup is more likely to over-represent counties with a higher percentage of individuals with a college degree than Twitter ($r = .39$ among Gallup counties, $r = .20$ among Twitter counties, $p < .001$). Twitter also over-represented counties with a higher black population ($r =.14$ among Twitter counties, $p < .001$). It is essential to consider these biases before making inferences about other populations, based on our findings.

Post-stratification. The populations of users in the Gallup and Twitter datasets are notably different, and potentially unrepresentative of the US population. Therefore, we tested the impact of post-stratification of both samples by age, gender, income, and education to match the county-level population distributions, as per the US Census (cf. (26)).

Post-stratification attempts to remove selection bias by taking a weighted average of individual-level responses, such that individuals are under (or over) represented in the sample are up (or down) weighted in the average (27–30). Weights are created by taking the ratio of a known population distribution (in this case, the US Census) to the sample distribution (in this case, Gallup and Twitter). If a particular auxiliary variable is under-represented in the sample, then the ratio (or weight) will be higher than one, in effect, treating this particular group of people as more important. Similarly, if the auxiliary variable is over-represented, then the weight will be less than one, and this group of people will be less important.

Both the county level Twitter and Gallup data were post-stratified using a raking algorithm across four auxiliary variables (age, gender, income, and education) (31). Raking is a widely used form of post-stratification, specifically used when correcting

for multiple auxiliary variables, and their full joint distribution is not known (31–34). In practice, full joint distributions are rarely known, even for a small number of auxiliary variables. In our case, information was unavailable for the full distribution of age × gender × income × education. The raking process iteratively estimates the full joint distribution using the marginal distributions for each of the auxiliary variables. For example, raking produces a joint distribution of age × income from an age distribution and an income distribution. It is important to note that these distributions are not continuous probability distributions, but rather percentages of the population within specific bins.

Gallup The known population distribution data was downloaded from the 2015 American Community Survey (5-year estimate) (35). While post-stratification was run independently for each county, we determined the number and size of bins by terciling national-level data for age, income, and education, and splitting gender into percentages of females and males. The national-level terciles gave us the final bin boundaries: *age* — 20 to 39, 40 to 54 and 55 or older; *income* — $0 to $34,999, $35,000 to $74,999 and $75,000 or higher; *education* — high school diploma or less, some college but less than a Bachelor's degree and a Bachelor's degree or higher.

For each participant in the Gallup survey, we had self-reported age, gender, income, and education. Age is a continuous variable, gender is binary (female/male), and both income and education are ordinals. The income ordinals were mapped to terciles as follows: $720 to $35,999 (to the first Census tercile; $0 to $34,999), $36,000 to $59,999 (to the second Census tercile; $35,000 to $74,999) and greater than $60,000 (to Census $75,000 or higher). The education ordinals mapped directly onto the census categories.

Twitter The Twitter sample data uses the County Tweet Lexical Bank (36). This dataset consists of roughly 6 million geo-located (to US counties) Twitter users. Each user posted at least 30 tweets, and each county contained at least 100 such users. For each Twitter user, we estimated age (continuous), gender (binary female/male), income (continuous), and education (binary below/above Bachelor's degree) from their tweet text (19, 26, 37). Accuracies of the language models are provided in Table S7.

Unlike with the Gallup data, we did not use National level terciles for the Twitter dataset. Instead, we used all bins as reported by the US Census (11 bins for age, 2 for gender, 10 for income, and 2 for education). Again, we used the 2015 American Community Survey (5-year estimate) as our known population data. To account for sparsity in our sample (i.e., socio-demographic bins in which none of our Gallup or Twitter users mapped), we used a minimum bin percentage threshold of 20%. That is, if a given bin did not contain at least 20% of our Twitter sample, the bin was combined with the adjacent bin. This process was repeated until all bins met the threshold or two bins remain. This was done independently for each county. As a result, each county was potentially post-stratified on a different number of bins.

Post-stratification bin percentages Tables S8a and S8b report average county bin percentages as reported from the US Census and our samples (Gallup and Twitter), before and after post-stratification. Since each auxiliary variable in the Gallup data set starts with at most three bins, we can easily calculate average county percentages, despite the binning process. On the other hand, the age and income Twitter data start with over ten bins, as opposed to three in the case of Gallup, which are collapsed independently across counties. Since each county post-stratifies on different numbers of age and income bins (between 2 and 10) and averages are calculated over a fixed bin size, we only report gender and education for Twitter. Full details of the Twitter post-stratification can be found in Giorgi et al. (26).

Table S9 summarizes the results of well-being prediction after post-stratification on the Gallup or Twitter data. The results are similar to the main results reported before stratification, both in direction and magnitude; the marginal effect of post-stratification suggests that our findings are robust.

The poststratification process relies heavily on accurately estimating sociodemographics from language. Noisy estimates about these factors can amplify errors in subsequent steps. This may occur due to 1) non-representative training data in the models and 2) regularization in the models, which will shrink the predicted distribution towards the mean of the training data. These issues were explored in (26). The authors showed that noisy person-level models led to a decrease in predictive performance at the county-level only when the number of demographics bins used in the post-stratification was large. Specifically, performance decreased when post-stratifying on age and income, both of which had at least ten demographic bins, whereas post-stratifying on gender and income (each with two bins) did not affect performance. In the current study, we used terciles for each of our four variables (age, gender, income, and education), which should have minimized the downstream effects of the noisy models. Note that the noisier the models, the harder the prediction task, that is, the lower the observed prediction accuracies. However, we did not observe significantly decreased performance with the terciled socio-demographic bins that made our training data more representative, alleviating concerns about excessive noise in the models.

Controlling for confounds

To test the robustness of our findings, we entered age, gender, state, and region dummies, as well as a socioeconomic index as covariate control variables into the language regressions. Table S10 summarizes the results of well-being prediction at the county-level, as a partial correlation controlling for region, age, and socioeconomic status. Region information was encoded as four census 'regions' (20) and 50 binary variables indicating the county's state. Age information comprised two variables denoting the percentage population under 18 years and the population over 65 years in the county. Socioeconomic status was encoded as the socioeconomic index described in Table S4.

Table S10 shows that patterns of language correlations were robust after controlling for demographic, and regional covariates, but largely did not account for variance in Life Satisfaction over and above socioeconomic status. Twitter is a strong direct

predictor of socioeconomic status in a cross-validation framework ($r = .85$ ($p < .001$) as in the last column of Table S5). Some of the word- and data-driven methods (e.g., LabMT, WWBP Affect, and Swiss Chocolate) capture variance in Happiness over and above socioeconomic status.

Stability over time

We examined whether the main findings replicate across two different periods: 2012-2013 and 2015-2016. For the years 2015-2016, we relied on additional data that was not a part of our initial dataset but was constructed the same way (a 10% random geotagged Twitter sample). First, we used the subset of Gallup and Twitter data which spanned 2012-2013, creating Gallup and language estimates across that time span. Next, we replicated our correlation analysis for 373 counties for which there was sufficient Gallup data and language available in 2012-2013, with the availability of the Gallup data limiting the data set more substantially. Next, we followed the same procedure data from 2015-2016. Finally, we compared the performance of language models trained on the 2012-2013 Twitter language which we applied to (and evaluated against) data from 2015-2016.

Table S11 summarizes the replication analysis performed on a subset of 373 counties from our main set of 1,208 counties. First, we reproduced the main results from Table 2 for comparison. We then reported the main results on a subset of 373 counties for which sufficient Twitter language was available in 2012-2013, and again in 2015-2016 (which is not included in our primary dataset). The results showed a pattern of correlations consistent with the main results, and across the two spans. Table S11 also shows that when predicting county well-being for a future time span (i.e., 2015-2016), the language models trained on 2012-2013 performed at par with language models trained on 2015-2016. This indicates that the changes in language use between 2013 and 2015 accounted for a very small difference in predictive performance, which lay within the confidence bounds of predicting with models trained on the language of the same year. These analyses suggest that our findings were robust at least across the time spans we were able to sample in this study.

Validation at the Individual Level

We evaluated whether our results replicated at the individual level – i.e., whether supervised models outperformed theory-based dictionaries for well-being prediction from social media language.

Data. We recruited adults in the United States to respond to a well-being survey via Qualtrics. This study was approved by the Institutional Review Board at the University of Pennsylvania. Our survey comprised demographic questions (age, gender, race, education, and income brackets as per the items in the National Census) and well-being items identical to the Gallup well-being questions (see Table S1). The question order was randomized. Our analysis is based on 2,321 individuals who consented to share their Facebook data and had posted at least 100 posts on Facebook. Summary statistics about the participants are provided in Table S12. By running ordinary least squares regression analysis between survey responses on well-being items and the language of Facebook posts, we validated the robustness of our findings at the user level and across two social media platforms.

Language features were derived using similar steps for tokenization and topic extraction as was carried out at the county level. Emotion measurements based on theory-based, word-level annotations, and post-level annotations were obtained and compared against survey-based language modeling using 2,000 topic features. In the case of the survey-based model, predictive performance was reported as the average Pearson's r on the held out observations in a ten-fold cross-validation setting (the *Direct prediction* column in Table S13), following the same feature selection and dimensionality reduction pipeline as applied at the county level.

Individual Level Results. Table S13 summarizes the results of well-being prediction at the individual level in the Qualtrics Facebook dataset. We see that trends similar to the county-level findings are observed in the Qualtrics Facebook dataset.

Overall, the best-performing model was the direct prediction model ($r = .26$, $p < .001$). Word-level methods, which were intended for person-level analyses, performed somewhat better at the individual level than at the county level, but LIWC's positive emotion dictionary had no significant correlation with survey-measured Happiness. The PERMA lexicon was better able to predict Life satisfaction at the individual level. A language prediction model based on 2,000 topic features from the language of 2,143 users was trained on the survey-reported Life satisfaction scores, validated on the held-out set of 178 users, and subsequently applied to the county-level as the 'person-level life satisfaction' model.

We conducted an error analysis at the individual level. We did not observe the same pattern of unexpected word correlations at the individual level as we did at the county level. This pattern suggests that the errors observed at the county-level may be mostly due to ecological influences across counties, due to socioeconomic gradients and cultural differences in language use.

Error Analysis

We conducted a posthoc diagnostic analysis of the word-level methods that focused on word correlations, highly frequent words, erroneous positive emotion words, and context effects.

Word correlations. We identified the most frequent words significantly correlated with Gallup Happiness ($p < 0.05$ with Benjamini-Hochberg correction). We devised a language confusion matrix to visualize the positive and negative words with correlations with Gallup Happiness opposite to expectation.

Highly frequent words. As dictionary frequencies are disproportionately determined by the most highly frequent words, we investigated if removing the most frequent words changed the pattern of correlations in the expected direction. For the LIWC 2015 positive emotion dictionary, we removed the three most frequent words that appeared on Twitter: 'lol,' 'love,' and 'good.' Weighted by valence, 'love' and 'good' were also the most frequent words in the positive part of the ANEW dictionary, so we removed these as well during the modification (it did not contain 'lol'). The positively-valenced part of the LabMT dictionary (valence > 6, following (7)) similarly contained 'lol,' 'love,' and 'good' among the most frequent – but we also observed that pronouns were included in the dictionary even after following Dodds et al. (7) in removing the words with valence 4 to 6. Subsequently, we used the LIWC pronoun dictionary to filter out pronouns, removing the following words: 'me,' 'we,' 'mine,' 'myself,' 'us,' 'you,' 'yours,' 'yourself,' 'she,' 'my,' 'herself,' 'our' in addition to 'lol,' 'love,' and 'good.' See Figure S3a for the correlations of these most frequent words with happiness across the three dictionaries, and Table S14 for its effect on improving well-being predictions. Additional supplementary materials on OSF provide figures showing the word composition of the dictionaries (weighted, where appropriate) in greater detail (38).

Mapping erroneous positive emotion words. We mapped the prevalence of the LIWC positive emotion words that correlated negatively with Happiness across the states of the US. In the absence of geographic confounds, the measurement errors would be uniformly distributed across the 50 states.

Context effects. As many words in the LIWC positive emotion dictionary also appear in other LIWC dictionaries, we used this overlap to study positive emotion words that also mark informal language, personal concerns, and social, perceptual, and biological processes. We again performed an ordinary least squares regression of the relative frequency of these sets of words against well-being, socioeconomic, and health variables.

Error Analysis Supplemental Results. Results for LIWC are reported in the main paper. Here we provide supplemental analyses and results that further identify errors that can occur with word-level approaches.

Figure S4 presents the language confusion matrices for the LabMT dictionary, treating the LabMT words with a score higher than 6 as positive words and words with a score less than 4 as negative words [ii]. The words along the diagonal correlated in the expected directions with county-level Happiness. Along the off-diagonal, the false LabMT positive words (top right) mostly comprised words referencing the self ('me'), family members ('baby,' 'daddy,' 'mommy,' and 'aunt'), and religion ('bless,' and 'faithful'). The false LabMT negative words (bottom left) included language reflecting political discourse ('political,' and 'conservatives'), finances ('taxes,' 'bill,' and 'mortgage'), and work ('delayed,' and 'deadline') which are negatively valenced when annotated at the word level but appeared to be used more frequently in the more affluent counties. As in the case of the LIWC dictionary, modifying the LabMT dictionary to remove some of the most frequent yet erroneous words (see Figure S3a) improved the county-level (see Table S3) and individual-level correlations with well-being items (see Table S13).

Race and cultural confounds affected the language-based predictions of well-being. Figure S3b shows how these confounds 'helped' the well-being prediction for common LIWC and LabMT true positive words; i.e., their usage along demographic and regional differences was mirrored in the differences in well-being. Figure S3c shows how these external biases can exacerbate the errors in frequently occurring LabMT positive emotion words; i.e., they were used differently by different communities, in ways which confound well-being measurements. For instance, in Figure S3c, we see that controlling for the percentage of African Americans in the population changed the association of 'lol' with well-being from $r = -.11$ ($p < .001$) to -.35 ($p < .001$).

Several other "true" LabMT negative words (Figure S4, bottom-right) (e.g., 'ni**a', 'ni**az', 'bi**hes') bore a racist or sexist connotation in general usage. However, within specific contexts, the words may have had different connotations. In colloquial usage, they may have connoted a friendly, familiar, or inclusive reference (39) when talking to or about others. Swear words (e.g., 'sh*t') may be used in a friendly manner to 'break the ice' in an informal conversation (40). Some appeared to signal 'Black Twitter' (41) through the playful modification of verb spellings (e.g., 'f**kin") using practices common in African American Vernacular English (42). Language differences appeared to reflect the socioeconomic and cultural differences that also explicate the differences in region-level well-being. Even as internet language keeps changing, the differences in language use can signal the persisting cultural and socioeconomic gaps in society.

In constructing dictionaries, annotators determine the connotation of words based on their most salient (not necessarily most frequent) word sense. However, their annotations may not correspond to the contemporary contextual usage of words or underlying psychological realities, in part because annotations inherently are impacted by the annotator's experiences and perspective. For instance, annotators for LabMT denoted 'me' as a word with high positive valence, but studies have found robust correlations between higher self-reference and poorer mental health, depression, and loneliness (43). Annotators recruited through online platforms (e.g., in the case of LabMT) are likely to be young, educated residents of liberal, urban areas in the US (44) which may explain why 'conservative' was annotated with a negative valence (LabMT, see Figure S4, bottom-left).

The results and the error analysis suggest that there may be a subset of LIWC positive emotion words that are significantly negatively correlated with the well-being and health measures. Table S15 deconstructs the LIWC positive emotion dictionary into other concepts, by referring to LIWC's dictionaries that also contain the same positive emotion words. While words within the positive emotion dictionary overlap with 49 other dictionaries, here we present the most salient results as examples of the impact of contextual effects, presenting dictionaries that represent informal language, personal concerns, and language that captures processes including social, perceptual, and biological processes.

[ii] We followed the authors' operationalization in (7)

Additional variable tables

- Table S4 provides the sources of data and any transformations that were performed on them.
- Table S17 provides the inter-item correlations among the dependent variables at the county- and the individual-level.
- Table S18 provides the inter-item correlations among the Gallup outcomes at the county-level, for 2012-2013 and 2015-2016 (N = 373 counties).
- Table S19 provides the inter-correlations among the measurements of other LIWC dictionaries, which also contain words from the LIWC positive emotion dictionary.
- Table S20 provides the inter-correlations between Twitter's emotion and well-being measurements, calculated at the county-level.

Fig. S1. Participant flow at the US county level for inclusion in the study.

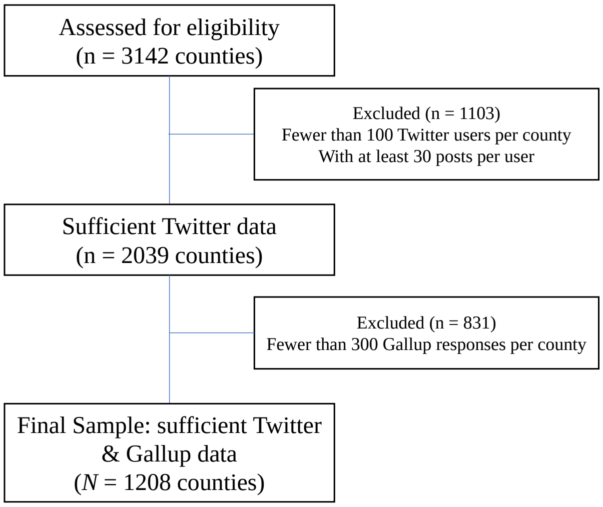

Fig. S2. Map of aggregated Gallup Life satisfaction scores for 1,208 US counties with at least 300 respondents.

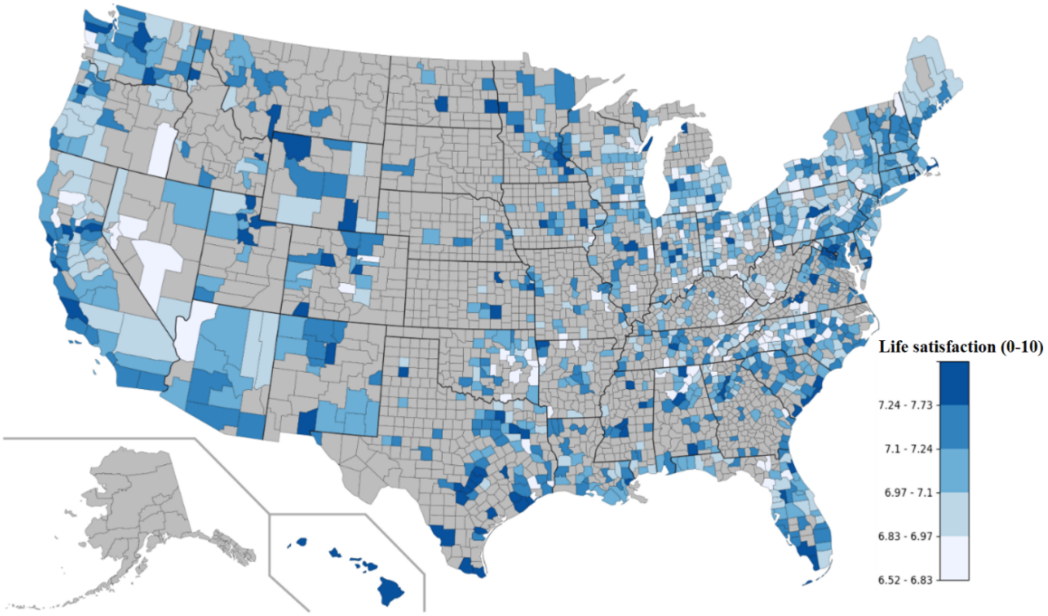

iii

Fig. S3. The association of the most frequent words in LIWC, ANEW, and LabMT with Gallup Happiness

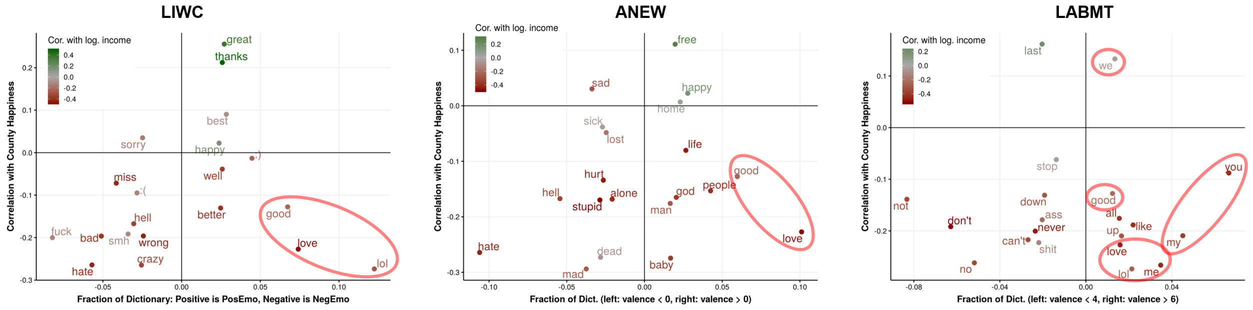

(a) Shown are the 10 most frequent words per positive and per negative side of LIWC (Positive and Negative Emotion dictionaries), ANEW (valence above and below the mean valence) and LabMT (valence > 6 as positive and < 4 as negative. Words with valence between 4 and 6 were removed following (7)). For ANEW and LabMT, words were weighted by their valence weights, LIWC has no weights on words. Position on the vertical axis reflects correlation with county Gallup Happiness. Red circles indicate the words removed during the modification of the dictionaries, which were among the most frequent words and were inversely correlated with Gallup Happiness. The color of the points shows that they also correlate inversely with log median household income.

N = 1208 counties Gallup Items	Most frequent True Positive words from LIWC and LabMT																								
	first (N = 14.2m)					fun (N = 7.8m)					great (N = 16.9m)					thanks (N = 16.2m)					weekend (N = 6.5m)				
	Controlling for:					*Controlling for:*					*Controlling for:*					*Controlling for:*					*Controlling for:*				
	-	*State*	*Age*	*Race*	*SES*	-	*State*	*Age*	*Race*	*SES*	-	*State*	*Age*	*Race*	*SES*	-	*State*	*Age*	*Race*	*SES*	-	*State*	*Age*	*Race*	*SES*
**Life Satisfaction**	.14	.20	.13	.22	.00	.18	.22	.19	.31	.03	.23	.27	.27	.29	-.02	.28	.33	.29	.32	-.05	.25	.31	.24	.26	.08
**Happiness**	.28	.25	.33	.28	.21	.31	.26	.32	.34	.24	.26	.24	.30	.24	.13	.21	.21	.26	.19	.03	.27	.26	.34	.26	.19
**Worry**	-.14	-.04	-.17	-.21	-.09	-.11	-.09	-.11	-.20	-.07	-.05	-.05	-.06	-.08	.04	-.02	-.06	-.05	-.04	.13	-.18	-.10	-.24	-.19	-.13
**Sadness**	-.28	-.19	-.29	-.32	-.17	-.27	-.23	-.27	-.34	-.15	-.22	-.19	-.24	-.22	.00	-.22	-.22	-.22	-.22	.07	-.29	-.25	-.32	-.29	-.16

(b) Correlations of the top 5 True Positive words (positive emotion words that correlate positively with Gallup Happiness), controlling for state and region, age, race, and socioeconomic covariates

N = 1208 counties Gallup Items	Most frequent False Negative words from LIWC and LabMT																								
	good (N = 42.2m)					like (N = 74.7m)					lol (N = 76.7m)					love (N = 46.4m)					me (N = 129.8m)				
	Controlling for:					*Controlling for:*					*Controlling for:*					*Controlling for:*					*Controlling for:*				
	-	*State*	*Age*	*Race*	*SES*	-	*State*	*Age*	*Race*	*SES*	-	*State*	*Age*	*Race*	*SES*	-	*State*	*Age*	*Race*	*SES*	-	*State*	*Age*	*Race*	*SES*
**Life Satisfaction**	-.09	-.09	-.18	-.17	.02	-.30	-.34	-.34	-.33	.00	-.11	-.17	-.18	-.35	.05	-.34	-.36	-.31	-.34	-.03	-.28	-.35	-.32	-.35	.04
**Happiness**	-.13	-.11	-.19	-.09	-.06	-.19	-.19	-.25	-.17	-.01	-.27	-.26	-.35	-.38	-.19	-.23	-.20	-.26	-.23	-.05	-.27	-.28	-.35	-.25	-.12
**Worry**	.02	.01	.02	.06	-.02	.02	.04	.04	.03	-.12	.10	.06	.11	.29	.05	.15	.10	.21	.15	.05	.05	.04	.08	.08	-.07
**Sadness**	.11	.04	.16	.10	.01	.21	.21	.23	.21	-.06	.25	.19	.30	.43	.12	.36	.29	.35	.36	.12	.29	.26	.33	.31	.04

(c) Correlations of the top False Negative words (positive emotion words that correlate negatively with Gallup Happiness), controlling for state and region, age, race, and socioeconomic covariates

Fig. S4. LabMT Language confusion matrix, indicating potential sources of error

Words from the LabMT dictionary measuring positive (valence > 6) and negative valence (valence < 4)
are plotted in different quadrants, with the size of the word denoting the magnitude of its correlation with Gallup's Happiness item (p<0.01 after Benjamini-Hochberg correction). The shade of the word depicts its normalized frequency, with darker shades reflecting higher frequencies relative to other words.
We refer to falsely correlating LabMT positive emotion words as false positives (top right) and to falsely correlating LabMT negative emotion words as false negatives (bottom left).

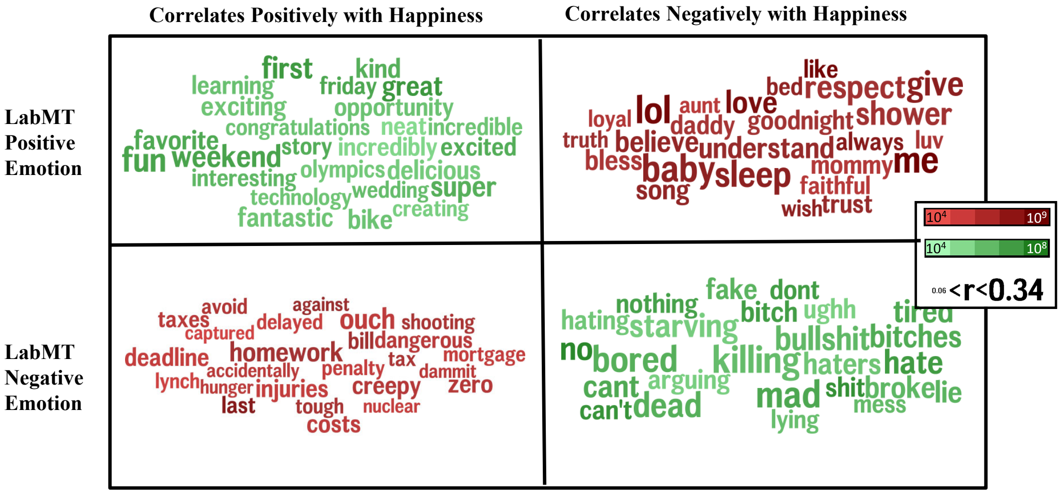

Table S1. Descriptive statistics for Gallup and Twitter users across 1,208 US counties.

(a) Descriptive statistics for the number of respondents, Twitter users, and Tweets by county.

	Per County					All 1208 Counties
**Data**	**Median**	**Mean**	**SD**	**Minimum**	**Maximum**	**Total**
Gallup respondents	692	1,429.8	2316.3	264	40,520	1,727,158
Twitter users	1004.5	4747.1	17,471.2	102	394,490	5,734,568
Tweets	190508	1,067,970.0	4,233,594.2	10,988	90,833,930	1,290,107,765

(b) Survey items included from the Gallup-Sharecare Well-Being Index, with the item description, scale, and mean scores across 1,208 counties.

Item Label	Description	Scale	Mean (SD)
Life satisfaction	Please imagine a ladder with steps numbered from zero at the bottom to ten at the top. The top of the ladder represents the best possible life for you, and the bottom of the ladder represents the worst possible life for you. On which step of the ladder would you say you personally feel you stand at this time?	0-10	6.97 (0.17)
	Did you experience the following feelings during A LOT OF THE DAY yesterday? How about -		
Happiness	Experienced happiness yesterday	Yes/No	0.89 (0.02)
Worry	Experienced worry yesterday	Yes/No	0.29 (0.03)
Sadness	Experienced sadness yesterday	Yes/No	0.17 (0.03)

Table S2. Twitter vs. Facebook language models, trained across the same sample of N = 522 qualtrics users. A comparison of the performance of a language model trained on Facebook vs. on Twitter language, which were used to derive county-level Twitter estimates of Life Satisfaction. Pearson's correlations with the Gallup well-being outcomes suggest that the difference between Facebook and Twitter is unlikely to have adversely affected the model performance of the Facebook-based WWBP Life Satisfaction model applied to county-level Twitter data in this study.

***N* = 1,208 U.S. counties**	**Person-level (Trained on N = 522 users)**			
	**Life Satisfaction**			
	Trained on Facebook language	[95% CI]	Trained on Twitter language	[95% CI]
Life Satisfaction	.38	[0.33, 0.43]	.33	[.28, .38]
Happiness	.25	[0.2, 0.3]	.22	[.17, .27]
Worry	-.04	[-0.1, 0.01]	-.02	[-.08, .04]
Sadness	-.27	[0.17, 0.27]	-.25	[-.30, -.19]

Table S3. Detailed predictive performance (reported as Pearson correlation) of the different types of language models: (a) word-level methods, (b) sentence-level methods, (c) person-level and direct-prediction methods.

(a) Performance of word-level methods

N = 1208 counties Gallup Items	Word-level: LIWC 2015											
	Positive	[95% CI]	Positive modified	[95% CI]	Negative	[95% CI]	Anger	[95% CI]	Anxiety	[95% CI]	Sadness	[95% CI]
Life Satisfaction	-.21	[-0.27, -0.16]	-.06	[-0.11, 0]	-.32	[-0.37, -0.27]	-.23	[-0.28, -0.18]	-.26	[-0.32, -0.21]	-.35	[-0.4, -0.3]
Happiness	-.13	[-0.18, -0.07]	.13	[0.07, 0.18]	-.27	[-0.32, -0.21]	-.27	[-0.32, -0.21]	-.07	[-0.12, -0.01]	-.07	[-0.12, -0.01]
Worry	.11	[0.06, 0.17]	.01	[-0.05, 0.07]	.03	[-0.03, 0.09]	.02	[-0.03, 0.08]	.07	[0.01, 0.12]	.00	[-0.05, 0.06]
Sadness	.25	[0.2, 0.3]	-.01	[-0.07, 0.04]	.22	[0.17, 0.28]	.17	[0.12, 0.23]	.16	[0.1, 0.21]	.18	[0.12, 0.23]

N = 1208 counties Gallup Items	Word-level: PERMA				ANEW				Warriner's		LabMT			
	Positive	[95% CI]	Negative	[95% CI]	Valence	[95% CI]	Valence modified	[95% CI]	Valence	[95% CI]	Valence	[95% CI]	Valence modified	[95% CI]
Life Satisfaction	.22	[0.16, 0.27]	-.37	[-0.42, -0.32]	-.03	[-0.09, 0.02]	.15	[0.1, 0.21]	.11	[0.05, 0.16]	-.27	[-0.32, -0.22]	.01	[-0.05, 0.07]
Happiness	.27	[0.22, 0.32]	-.17	[-0.22, -0.11]	.04	[-0.02, 0.1]	.18	[0.12, 0.23]	.18	[0.13, 0.24]	-.07	[-0.13, -0.02]	.16	[0.1, 0.21]
Worry	-.01	[-0.06, 0.05]	.02	[-0.04, 0.08]	.03	[-0.03, 0.09]	-.05	[-0.1, 0.01]	-.09	[-0.15, -0.04]	.02	[-0.04, 0.07]	-.04	[-0.09, 0.02]
Sadness	-.19	[-0.25, -0.14]	.18	[0.12, 0.23]	.09	[0.04, 0.15]	-.10	[-0.16, -0.04]	-.17	[-0.22, -0.11]	.19	[0.14, 0.25]	-.09	[-0.14, -0.03]

(b) Performance of sentence-level methods

N = 1208 counties Gallup Items	Sentence-level: NRC #Hashtag Emotion														WWBP Affect		Swiss Chocolate			
	Anticipation	[95% CI]	Joy	[95% CI]	Surprise	[95% CI]	Trust	[95% CI]	Fear	[95% CI]	Sadness	[95% CI]	Anger	[95% CI]	Disgust	[95% CI]	Affect	[95% CI]	Positive	[95% CI]	Negative	[95% CI]
Life Satisfaction	.38	[0.33, 0.43]	.21	[0.15, 0.26]	.30	[0.24, 0.35]	-.16	[-0.21, -0.1]	.30	[0.25, 0.36]	-.11	[-0.16, -0.05]	-.29	[-0.35, -0.24]	-.19	[-0.24, -0.13]	.29	[0.24, 0.34]	.24	[0.19, 0.3]	-.29	[-0.34, -0.24]
Happiness	.23	[0.18, 0.28]	.21	[0.15, 0.26]	.24	[0.18, 0.29]	-.19	[-0.25, -0.14]	.25	[0.2, 0.3]	-.20	[-0.25, -0.14]	-.24	[-0.29, -0.19]	-.18	[-0.24, -0.13]	.23	[0.18, 0.29]	.24	[0.19, 0.29]	-.30	[-0.35, -0.25]
Worry	-.04	[-0.1, 0.01]	-.01	[-0.07, 0.04]	-.05	[-0.11, 0.01]	.02	[-0.04, 0.07]	-.06	[-0.12, -0.01]	.00	[-0.06, 0.05]	-.01	[-0.07, 0.04]	-.05	[-0.1, 0.01]	.00	[-0.06, 0.06]	-.02	[-0.08, 0.03]	.11	[0.06, 0.17]
Sadness	-.29	[-0.34, -0.24]	-.13	[-0.18, -0.07]	-.32	[-0.37, -0.27]	.25	[0.2, 0.31]	-.29	[-0.34, -0.24]	.20	[0.14, 0.25]	.19	[0.13, 0.24]	.12	[0.06, 0.17]	-.18	[-0.23, -0.12]	-.20	[-0.25, -0.14]	.33	[0.27, 0.37]

(c) Performance of person-level and direct-prediction methods

N = 1208 counties Gallup Items	Person-level		Direct prediction							
	WWBP Life Satisfaction	[95% CI]	SES	[95% CI]	All LIWC dictionaries	[95% CI]	All language	[95% CI]	All language + SES	[95% CI]
Life Satisfaction	.39	[0.34, 0.44]	.59	[.55, .62]	.55	[.51, .59]	.62	[.58, .66]	.65	[.61, .69]
Happiness	.23	[0.17, 0.28]	.35	[.29, .39]	.48	[.43, .53]	.51	[.47, .54]	.52	[.48, .56]
Worry	-.03	[-0.09, 0.02]	-.21	[-.26, -.15]	.46	[.41, .51]	.52	[.48, .56]	.53	[.49, .57]
Sadness	-.23	[-0.28, -0.18]	-.50	[-.54, -.46]	.58	[.54, .62]	.64	[.60, .68]	.65	[.61, .69]

a. LIWC is Linguistic Inquiry & Word Count: Positive and Negative emotion, Anger, Anxiety, Sadness
ANEW is Affective Norms of English Words: Valence scores

b. NRC Hashtag Emotion: Anticipation, Joy, Surprise, Fear, Sadness, Anger and Disgust scores WWBP Affect: Affect
Swiss Chocolate: Positive, Negative scores

Table S4. Variable Sources and Transformation.

Variable			Description of variable	Unit	Years covered	Source
Included variable	Transformation	Categories				
% Female	-	Demographic	Percent female population	% of population	2010-2015	American Community Survey (ACS, 2015) 5-Year Estimates (5)
% Under 18			Percent population under 18 years			
% Over 65			Percent population over 65 years			
% African American			Percent African American population			
% Hispanic			Percent Hispanic population			
% Rural			Percentage of the county which is considered rural			
Income	log-transformed	Income	Median per capita income	2010 inflation-adjusted US dollars		
Household income	log-transformed		Median household income	2010 inflation-adjusted US dollars		
% with Bachelor's degree	-	Education	Percent population who attained a Bachelor's degree or higher	% of population		
Socioeconomic index	Independently standardized and then averaged	Income	Log-transformed per-capita income	% of population		
		Education	Attainment of bachelor's degree or higher			
% Fair/poor health		Health	Adults (age 18+) reporting fair or poor health	% of population		County-level estimates based on CDC's Behavioral Risk Factor Surveillance System (BRFSS) data (2009-2010) (6), obtained through 2013 County Health Rankings (CHR; 2015) (7)
Mentally unhealthy days			Adults (age 18+) reporting mentally unhealthy days			
All-cause mortality				per 100,000 population	2009-2016	CDC Wonder, Underlying Cause of Death (CDC, 2016) (8)

Table S5. Performance for the county-level prediction of demographic and health factors, reported as Pearson's correlation against the major Twitter-based methods for emotion and language modeling. Cells are shaded according to the strength of the positive or negative correlation after Benjamini-Hochberg correction

(a) Performance of word-level methods

N = 1208 counties Gallup Items	Word-level																
	LIWC						PERMA				ANEW				LabMT			
	Positive	[95% CI]	Positive modified	[95% CI]	Negative	[95% CI]	Positive	[95% CI]	Negative	[95% CI]	Valence	[95% CI]	Valence modified	[95% CI]	Valence	[95% CI]	Valence modified	[95% CI]
SES Index	-.40	[-0.45, -0.35]	-.08	[-0.14, -0.03]	-.48	[-0.53, -0.44]	.39	[0.34, 0.43]	-.43	[-0.47, -0.38]	-.12	[-0.18, -0.07]	.18	[0.12, 0.23]	-.43	[-0.48, -0.38]	.07	[0.02, 0.13]
% Fair/poor health	.37	[0.32, 0.42]	-.03	[-0.09, 0.03]	.35	[0.3, 0.4]	-.34	[-0.39, -0.29]	.19	[0.14, 0.25]	.11	[0.05, 0.17]	-.13	[-0.19, -0.08]	.25	[0.19, 0.3]	-.19	[-0.24, -0.13]
All cause mortality	.26	[0.21, 0.31]	-.14	[-0.2, -0.09]	.42	[0.37, 0.47]	-.38	[-0.43, -0.33]	.35	[0.3, 0.4]	.12	[0.07, 0.18]	-.14	[-0.2, -0.09]	.32	[0.27, 0.37]	-.17	[-0.22, -0.11]
Mentally unhealthy days	.19	[0.14, 0.25]	.01	[-0.05, 0.07]	.17	[0.12, 0.23]	-.14	[-0.2, -0.09]	.16	[0.1, 0.21]	.11	[0.05, 0.16]	-.04	[-0.09, 0.02]	.18	[0.13, 0.24]	-.04	[-0.09, 0.02]

(b) Performance of data-driven methods

N = 1208 counties Gallup Items	Sentence-level						Person-level		Direct prediction	
	WWBP		Swiss Chocolate							
	Affect	[95% CI]	Positive	[95% CI]	Negative	[95% CI]	WWBP Life Satisfaction	[95% CI]	All language	[95% CI]
SES Index	.39	[0.34, 0.44]	.40	[0.35, 0.45]	-.53	[-0.57, -0.49]	.54	[0.5, 0.58]	.85	[.80, .90]
% Fair/poor health	-.26	[-0.31, -0.21]	-.33	[-0.38, -0.28]	.52	[0.47, 0.56]	-.32	[-0.37, -0.27]	.75	[.73, .77]
All cause mortality	-.38	[-0.43, -0.34]	-.45	[-0.5, -0.41]	.51	[0.46, 0.55]	-.39	[-0.44, -0.35]	.82	[.80, .84]
Mentally unhealthy days	-.14	[-0.19, -0.08]	-.15	[-0.2, -0.09]	.25	[0.19, 0.3]	-.21	[-0.26, -0.15]	.51	[.46, .56]

Table S6. Summary of the potential sample biases caused by the absence of some counties in our dataset. Negative Pearson correlations indicate that counties with a given demographic feature are more likely to be missing in the datasets.

	Correlation with inclusion		
	Gallup	Twitter	Current dataset
	1,228 counties	2,039 counties	1,208 counties
% Population under 18	.01	.08	.02
% Population over 65	-.30	-.36	-.29
% African-American	.02	.14	.02
% Hispanic	-.06	-.13	-.06
% Male	-.20	-.22	-.20
% Rural	-.61	-.60	-.61
% Bachelor's degree	.39	.20	.38
Per capita income	.32	.20	.31

This Table shows the sample biases of the Gallup, Twitter and combined datasets as correlations against an dummy variable marking inclusion. Rural counties are especially underrepresented.

Table S7. Performance of user level models used to predict the sociodemographic labels for county-level tweets

		N	Test Accuracy
Age	Sap et al. 2014 (19)	75,394	.83 (Pearson r)
Gender			.92 (Accuracy)
Income	Matz et al. 2019 (37)	2,623	.41 (Pearson r)
Education	Giorgi et al. 2019 (26)	4,062	.62 / .53 (Accuracy / F1)

Table S8. Dataset statistics pre- and post-stratification as compared to the census demographic distribution.

(a) Average county bin percentages from the Census, Gallup and post-stratified Gallup.

	Age			Gender		Income			Education		
	18-39	*40-54*	*55+*	*Female*	*Male*	*$0-$34,999*	*$35,000-$74,999*	*$75,000+*	*High School equiv. or lower*	*Some college*	*Bach. Degree +*
**Census**	34.5	27.2	38.3	50.6	49.4	35.3	32.9	31.8	44.0	30.6	25.4
**Gallup**	22.4	25.1	52.5	49.6	50.4	37.7	24.1	38.2	28.8	32.6	38.6
**Post-stratified Gallup**	34.5	27.2	38.3	50.6	49.4	35.3	32.9	31.8	44.0	30.6	25.4

(b) Average county bin percentages from the Census, Twitter and post-stratified Twitter.

	Gender		Education	
	Female	*Male*	*Less than Bach. Degree*	*Bach. Degree or higher*
**Census**	50.6	49.4	74.6	25.4
**Twitter**	52.2	47.8	58.2	41.8
**Post-stratified Twitter**	50.6	49.4	74.6	25.4

Table S9. Summary of the best performing language models after the post-stratification of Twitter and Gallup data. The results are similar to those obtained before age, gender, income, and education post-stratification.

(a) Performance of word-level methods

N = 1208 counties Gallup Items	Word-level															
	LIWC						PERMA				ANEW				LabMT			
	Positive	[95% CI]	Positive modified	[95% CI]	Negative	[95% CI]	Positive	[95% CI]	Negative	[95% CI]	Valence	[95% CI]	Valence modified	[95% CI]	Valence	[95% CI]	Valence modified	[95% CI]
**Life Satisfaction**	-.24	[-0.29, -0.18]	-.02	[-0.07, 0.04]	-.33	[-0.38, -0.28]	.26	[0.21, 0.31]	-.28	[-0.33, -0.23]	-.09	[-0.15, -0.04]	.08	[0.02, 0.14]	-.29	[-0.34, -0.24]	.02	[-0.04, 0.08]
**Happiness**	-.13	[-0.18, -0.07]	.15	[0.1, 0.21]	-.30	[-0.35, -0.25]	.31	[0.26, 0.36]	-.14	[-0.19, -0.08]	-.02	[-0.08, 0.03]	.11	[0.06, 0.17]	-.12	[-0.18, -0.07]	.13	[0.08, 0.19]
**Worry**	.16	[0.1, 0.21]	.05	[-0.01, 0.11]	.06	[0, 0.12]	-.02	[-0.08, 0.03]	.03	[-0.03, 0.09]	.09	[0.03, 0.14]	.00	[-0.06, 0.05]	.12	[0.07, 0.18]	.03	[-0.03, 0.08]
**Sadness**	.25	[0.2, 0.31]	-.04	[-0.09, 0.02]	.28	[0.23, 0.33]	-.25	[-0.3, -0.2]	.17	[0.11, 0.22]	.11	[0.05, 0.16]	-.07	[-0.12, -0.01]	.23	[0.17, 0.28]	-.09	[-0.14, -0.03]

(b) Performance of data-driven methods

N = 1208 counties Gallup Items	Sentence-level						Person-level		Direct prediction	
	WWBP		Swiss Chocolate							
	Affect	[95% CI]	Positive	[95% CI]	Negative	[95% CI]	WWBP Life Satisfaction	[95% CI]	All language	95% CI
**Life Satisfaction**	.22	[0.17, 0.27]	.26	[0.21, 0.31]	-.44	[-0.48, -0.39]	.40	[0.35, 0.44]	.59	[.55, .63]
**Happiness**	.12	[0.07, 0.18]	.28	[0.22, 0.33]	-.39	[-0.44, -0.34]	.25	[0.2, 0.3]	.49	[.46, .52]
**Worry**	.00	[-0.06, 0.06]	-.04	[-0.1, 0.01]	.17	[0.22, 0.11]	-.08	-0.14, -0.02	.46	[.42, .50]
**Sadness**	-.12	[-0.18, -0.07]	-.25	[-0.31, -0.2]	.43	[0.47, 0.38]	-.27	-0.32, -0.22	.61	[.57, .64]

Table S10. Summary of the best performing language models as partial correlations controlling for the effect of region and state, age, race, and socioeconomic differences. The first column in each block provides Pearson's r with no controls.

(a) Partial correlations with word-level methods

N = 1208 counties Gallup Items	Word-level																								
	LIWC 2015															PERMA									
	Controlling for:					*Controlling for:*					*Controlling for:*					*Controlling for:*					*Controlling for:*				
	-	*State + Region*	*Age*	*Race*	*SES*	-	*State + Region*	*Age*	*Race*	*SES*	-	*State + Region*	*Age*	*Race*	*SES*	-	*State + Region*	*Age*	*Race*	*SES*	-	*State + Region*	*Age*	*Race*	*SES*
	Positive					Positive modified					Negative					Positive					Negative				
**Life Satisfaction**	-.21	-.26	-.22	-.21	.02	-.06	-.06	.00	-.02	-.01	-.32	-.34	-.33	-.38	-.05	.22	.28	.24	.35	-.01	-.37	-.36	-.35	-.37	-.15
**Happiness**	-.13	-.13	-.18	-.13	.01	.13	.10	.14	.08	.16	-.27	-.25	-.31	-.25	-.13	.27	.26	.31	.28	.16	-.17	-.16	-.18	-.19	-.03
**Worry**	.11	.05	.16	.11	.04	.01	-.02	.03	-.03	-.01	.03	.03	.05	.05	-.09	-.01	-.02	-.01	-.05	.08	.02	.03	.03	.01	-.08
**Sadness**	.25	.18	.26	.25	.06	-.01	-.01	-.06	.03	-.05	.22	.22	.23	.23	-.02	-.19	-.19	-.21	-.22	.00	.18	.19	.16	.19	-.04

(b) Partial correlations with word-level methods (continued)

N = 1208 counties Gallup Items	Word-level																			
	LabMT										ANEW									
	Controlling for:					*Controlling for:*					*Controlling for:*					*Controlling for:*				
	-	*State + Region*	*Age*	*Race*	*SES*	-	*State + Region*	*Age*	*Race*	*SES*	-	*State + Region*	*Age*	*Race*	*SES*	-	*State + Region*	*Age*	*Race*	*SES*
	Valence					Valence modified					Valence					Valence modified				
**Life Satisfaction**	-.27	-.25	-.23	-.27	-.03	.01	.03	.07	.04	-.03	-.03	-.03	.00	-.03	.04	.15	.16	.17	.16	.05
**Happiness**	-.07	-.06	-.07	-.08	.09	.16	.14	.21	.13	.13	.04	.07	.05	.03	.08	.18	.18	.20	.16	.12
**Worry**	.02	.01	.05	.02	-.09	-.04	-.02	-.03	-.06	-.02	.03	.02	.05	.03	.01	-.05	-.03	-.04	-.05	-.01
**Sadness**	.19	.15	.16	.20	-.03	-.09	-.05	-.13	-.07	-.05	.09	.02	.05	.10	.03	-.10	-.13	-.12	-.09	-.01

(c) Partial correlations with sentence-level methods

N = 1208 counties Gallup Items	Sentence-level														
	WWBP Affect					Swiss Chocolate									
	Controlling for:					*Controlling for:*					*Controlling for:*				
	-	*State + Region*	*Age*	*Race*	*SES*	-	*State + Region*	*Age*	*Race*	*SES*	-	*State + Region*	*Age*	*Race*	*SES*
	Affect					Positive					Negative				
**Life Satisfaction**	.29	.31	.29	.35	.08	.24	.28	.29	.34	.01	-.29	-.37	-.36	-.40	.03
**Happiness**	.23	.23	.25	.21	.11	.24	.23	.29	.23	.12	-.30	-.31	-.42	-.31	-.17
**Worry**	.00	-.03	.00	-.02	.10	-.02	-.03	-.03	-.06	.07	.11	.07	.16	.17	.00
**Sadness**	-.18	-.21	-.17	-.18	.02	-.19	-.19	-.23	-.21	.00	.33	.28	.38	.37	.08

(d) Partial correlations with person-level methods

N = 1208 counties Gallup Items	Person-level				
	WWBP Life Satisfaction				
	Controlling for:				
	-	*State + Region*	*Age*	*Race*	*SES*
**Life Satisfaction**	.39	.40	.39	.39	.11
**Happiness**	.23	.23	.26	.22	.05
**Worry**	-.03	-.05	-.06	.03	.11
**Sadness**	-.23	-.24	-.22	-.23	.06

Table S11. Summary of the replication analyses (N = 373 counties).

(a) The main results from Table 2: Pearson correlations of the Twitter language from 2009-2015 and the Gallup-Sharecare well-being estimates, N = 1,208 US counties

2009 - 2015 N = 1208 counties Gallup Items	Word-level									Sentence-level			Person-level	Direct prediction
	LIWC			PERMA		ANEW		LabMT		WWBP	Swiss Chocolate		WWBP Life Satisfaction	All language
	Positive	Positive (modified)	Negative	Positive	Negative	Valence	Valence (modified)	Valence	Valence (modified)	Affect	Positive	Negative		
Life Satisfaction	-.21	-.06	-.32	.22	-.37	-.03	.15	-.27	.01	.29	.24	-.29	.39	.62
Happiness	-.13	.13	-.27	.27	-.17	.04	.18	-.07	.16	.23	.24	-.30	.23	.51
Worry	.11	.01	.03	-.01	.02	.03	-.05	.02	-.04	.00	-.02	.11	-.03	.52
Sadness	.25	-.01	.22	-.19	.18	.09	-.10	.19	-.09	-.18	-.20	.33	-.23	.64

(b) Subset of the main results: Pearson correlations of the Twitter language from 2009-2015 and the Gallup-Sharecare well-being estimates, N = a subset of 373 US counties with sufficient language and Gallup responses (> 200) for 2012-2013 and 2015-2016.

2009 - 2015 N = 373 counties Gallup Items	Word-level									Sentence-level			Person-level	Direct prediction
	LIWC			PERMA		ANEW		LabMT		WWBP	Swiss Chocolate		WWBP Life Satisfaction	All language
	Positive	Positive (modified)	Negative	Positive	Negative	Valence	Valence (modified)	Valence	Valence (modified)	Affect	Positive	Negative		
Life Satisfaction	-.17	.02	-.38	.28	-.39	.01	.13	-.24	.00	.32	.31	-.33	.43	.64
Happiness	.01	.34	-.36	.44	-.18	.31	.39	.12	.32	.36	.29	-.32	.28	.72
Worry	.01	-.05	.07	-.12	.06	-.24	-.27	-.20	-.20	-.04	.00	.08	-.07	.64
Sadness	.16	-.08	.26	-.30	.16	-.11	-.25	.01	-.20	-.19	-.17	.31	-.28	.68

(c) Replication analysis 2012-2013: Pearson correlations of the Twitter language and the Gallup-Sharecare well-being estimates on N = the same 373 US counties as in (b).

2012 - 2013 N = 373 counties Gallup Items	Word-level									Sentence-level			Person-level	Direct prediction
	LIWC			PERMA		ANEW		LabMT		WWBP	Swiss Chocolate		WWBP Life Satisfaction	All language
	Positive	Positive (modified)	Negative	Positive	Negative	Valence	Valence (modified)	Valence	Valence (modified)	Affect	Positive	Negative		
Life Satisfaction	-.16	-.07	-.36	.23	-.29	-.04	.02	-.23	-.11	.31	.32	-.07	.36	.47
Happiness	.10	.29	-.26	.33	-.15	.12	.19	.09	.20	.35	.24	-.02	.15	.60
Worry	-.11	-.12	.02	-.06	.03	-.14	-.16	-.16	-.16	-.06	-.01	-.06	.03	.38
Sadness	.03	-.05	.18	-.23	.10	-.11	-.17	-.04	-.15	-.15	-.14	-.03	-.20	.51

(d) Replication analysis 2015-2016: Pearson correlations of the Twitter language and the Gallup-Sharecare well-being estimates on N = the same 373 US counties as in (b), and (c).

2015 - 2016 N = 373 counties Gallup Items	Word-level									Sentence-level			Person-level	Direct prediction
	LIWC			PERMA		ANEW		LabMT		WWBP	Swiss Chocolate		WWBP Life Satisfaction	All language
	Positive	Positive (modified)	Negative	Positive	Negative	Valence	Valence (modified)	Valence	Valence (modified)	Affect	Positive	Negative		
Life Satisfaction	-.08	.08	-.32	.22	-.18	-.10	-.03	-.11	-.14	.35	.31	-.27	.39	.54
Happiness	.03	.17	-.32	.24	-.10	.03	.12	-.03	-.07	.33	.30	-.17	.28	.44
Worry	.08	.06	.10	-.02	.03	-.07	-.13	.04	.05	-.09	-.06	-.01	-.14	.46
Sadness	.09	-.05	.27	-.15	.07	.06	-.04	.12	.06	-.24	-.23	.24	-.29	.30

(e) Robustness analysis to compare predictive performances of language models trained on the Twitter language of 2012-2013 and 2015-2016 respectively. The test set comprised the language for N = 373 counties in 2015-2016. We see that the predictive performance of 2012-2013 language models performed close to those trained on the same year, suggesting that our language analyses are robust over time.

N = 373 counties Gallup Items from 2015 - 2016	Language models from Twitter (2012- 2013)		Language models from Twitter (2015- 2016)	
	Direct prediction	[95% CI]	Cross-validated	[95% CI]
Life Satisfaction	.47	[.37, .53]	.54	[.46, .62]
Happiness	.43	[.33, .49]	.44	[.35, .51]
Worry	.42	[.29, .46]	.46	[.38, .54]
Sadness	.29	[.27, .55]	.30	[.28, .46]

Table S12. Summary statistics about the Qualtrics dataset of individual Facebook users (N = 2,321), reported as demographic information about the survey respondents and scales used to measure subjective well-being.

(a) Statistics for the Qualtrics dataset.

N	Mean Age (SD)	% Female
2,321	38.5 (18.6)	61.6%

(b) Survey items and descriptive statistics for the dependent variables in the Qualtrics dataset.

Item Label	Facebook users (N = 2321)	
	Scale	Mean (SD)
Life satisfaction	0-10	6.04 (2.22)
Happiness		6.17 (2.76)
Worry	0-10	4.49 (3.04)
Sadness		3.40 (3.13)

Table S13. Correlations at the individual level between self-reported well-being and language-based well-being estimates across a Qualtrics sample of N = 2,321 participants. Participants answered the same survey questions as the Gallup sample (but for happiness, worry and sadness on continuous 0-10 scales as opposed to yes/no.)

Facebook users (N = 2321)	Word-level															
	LIWC						PERMA				ANEW				LabMT			
	Positive	[95% CI]	Positive modified	[95% CI]	Negative	[95% CI]	Positive	[95% CI]	Negative	[95% CI]	Valence	[95% CI]	Valence modified	[95% CI]	Valence	[95% CI]	Valence modified	[95% CI]
Life Satisfaction	.04	[0, 0.08]	.07	[0.03, 0.11]	-.26	[-0.29, -0.22]	.14	[0.1, 0.18]	-.21	[-0.25, -0.17]	.09	[0.05, 0.13]	.12	[0.08, 0.16]	-.02	[-0.06, 0.02]	.00	[-0.04, 0.04]
Happiness	.04	[0, 0.08]	.06	[0.02, 0.1]	-.21	[-0.25, -0.17]	.11	[0.07, 0.15]	-.18	[-0.22, -0.14]	.08	[0.04, 0.12]	.09	[0.05, 0.13]	.00	[-0.04, 0.04]	.00	[-0.04, 0.05]
Worry	.07	[0.03, 0.11]	.05	[0.01, 0.09]	.13	[0.09, 0.17]	.00	[-0.04, 0.04]	.15	[0.11, 0.19]	.03	[-0.01, 0.07]	.00	[-0.04, 0.04]	.08	[0.04, 0.12]	.05	[0.01, 0.09]
Sadness	.04	[0, 0.08]	.01	[-0.03, 0.05]	.15	[0.11, 0.19]	-.04	[-0.08, 0]	.14	[0.1, 0.18]	.01	[-0.03, 0.05]	-.02	[-0.06, 0.02]	.07	[0.02, 0.11]	.03	[-0.01, 0.07]

Facebook users (N = 2321)	Sentence-level						Direct prediction	
	WWBP		Swiss Chocolate					
	Affect	[95% CI]	Positive	[95% CI]	Negative	[95% CI]	All language	[95% CI]
Life Satisfaction	.22	[0.19, 0.26]	.21	[0.17, 0.25]	-.12	[-0.16, -0.08]	.26	[.22, .30]
Happiness	.20	[0.16, 0.24]	.15	[0.11, 0.19]	-.07	[-0.11, -0.03]	.15	[.11, .19]
Worry	-.07	[-0.11, -0.03]	-.08	[-0.12, -0.04]	.07	[0.03, 0.12]	.17	[.15, .19]
Sadness	-.10	[-0.14, -0.06]	-.10	[-0.14, -0.06]	.08	[0.04, 0.12]	.17	[.13, .21]

Table S14. Impact of removing the frequent, erroneous words driving LIWC and LabMT correlations.

N = 1208 counties<br>Gallup Items	Post-modification results					
	LIWC		ANEW		LabMT	
	Positive	Positive modified	Valence	Valence modified	Valence	Valence modified
Life Satisfaction	-.21	-.06	-.03	.15	-.27	.01
Happiness	-.13	.13	.04	.18	-.07	.16
Worry	.11	.01	.03	-.05	.02	-.04
Sadness	.25	-.01	.09	-.10	.19	-.09
Socioeconomic index	-.40	-.08	-.12	.18	-.43	.07
% Fair/poor health	.37	-.03	.11	-.13	.25	-.19
All cause mortality	.26	-.14	.12	-.14	.32	-.17
Mentally unhealthy days	.19	.01	.11	-.04	.18	-.04

Table S15. Overlap of LIWC 2015 positive emotion words with other LIWC dictionaries.

LIWC 2015 dictionaries	Informal Language			Personal Concerns						
	Swear	Assent	Netspeak	Religion	Leisure	Work	Money	Social	Perceptual	Biological
Most frequent positive emotion words	lmao*, lmfao*	ok, cool, awesome, okay, yay*	lol, :), haha*, lmao*, lmfao*	bless*, faith*, heaven*, worship*, paradise*	play, fun, party*, playing, joke*	champ*, award*, success, challeng*, credit*	free, credit*, rich, charit*, profit*	love, party*, welcom*, trust*, giving*	cool, beautiful, laugh*, sweet, warm	love, sweet, sexy, loved, loves
Life Satisfaction	-.04	-.04	-.13	-.11	.15	.33	.23	-.32	-.02	-.32
Happiness	-.27	-.01	-.25	-.12	.15	.23	.12	-.17	.14	-.20
Worry	.12	-.02	.10	.08	-.04	-.05	-.02	.12	-.03	.14
Sadness	.14	.02	.23	.27	-.21	-.30	-.17	.32	-.02	.34
Socioeconomic index	-.05	-.09	-.33	-.33	.26	.57	.40	-.50	-.06	-.53
% Fair/poor health	.21	.09	.42	.43	-.25	-.44	-.27	.37	-.06	.40
All cause mortality	.11	.07	.30	.49	-.22	-.48	-.41	.38	-.14	.40
Mentally unhealthy days	.07	.06	.15	.24	-.13	-.23	-.15	.25	.01	.27
Words in positive emotion dictionary	2	9	18	6	24	17	14	59	30	25
Fraction of positive emotion word occurrences (tokens)	3.70%	5.38%	26.58%	1.06%	6.57%	1.71%	2.32%	14.15%	4.42%	9.67%

Table S16. The complete set of language-based emotion measures used in this study, including the ones reported on in Table 2 and Table S3. The number of features differs slightly from their intended sizes since we did not include multi-word phrases in the LIWC dictionaries and part-of-speech tags in the Swiss Chocolate model.

	Method (source)	Number of features (words)	Categories	Development
Word-level annotations	**LIWC 2015** (2)	1,364	Positive Emotion, Negative Emotion, Anxiety, Anger, Sadness	Developed by psychologists to measure the psycholinguistic concepts elicited in an individual's speech and writing
	**PERMA dictionary** (3, 4)	402	Positive Emotion, Negative Emotion	Developed by psychologists based on Seligman's theory of well-being. Version 2: manually defined.
	**ANEW** (5)	1,034	Valence	Annotation experiments for words, based on a 7-point Likert scale
	**Warriner's extended ANEW** (6)	13,905	Valence	Annotation experiments for words, based on a 7-point Likert scale
	**LabMT** (7)	10,218	Valence	Annotation experiments for words, based on a 1-9 scale for valence. Following (7), after removing words with 4 < valence < 6, a total of 3,731 words remain in our analyses.
Sentence-level annotations	**WWBP Affect** (12)	7,265	Affect	Standardized regression coefficients for words, inferred from supervised machine learning models trained on labeled Twitter posts
	**Swiss Chocolate** (9)	7,168	Positive, Neutral, and Negative Emotion	Standardized regression coefficients for words, inferred from supervised machine learning models trained on hashtagged Twitter posts
	**NRC Hashtag Emotion** (8)	16,862	Anticipation, Joy, Surprise, Trust, Fear, Sadness, Anger, Disgust	Standardized regression coefficients for words, inferred from supervised machine learning models trained on hashtagged Twitter posts
Person-level models	**Life Satisfaction** (This study)	2,000 LDA topics	Cantril Ladder	Standardized regression coefficients inferred from supervised machine learning models trained on the social media posts of 2,143 survey respondents
Direct prediction	**All LIWC dictionaries** (2)	6,549	Emotion concepts, cognitive processes, personal concerns and other dictionaries of psycholinguistic relevance.	Standardized regression coefficients for LIWC categories, inferred from supervised machine learning models that are trained on the county's relative usage of LIWC categories when its well-being measurement is known
	**County Life Satisfaction** (This study)	2,000 LDA topics	Cantril Ladder	Standardized regression coefficients inferred from supervised machine learning models trained on the social media posts of 1208 counties

For LabMT, we followed (7) in removing 'neutral' words with $4 < \text{valence} < 6$, leaving 3,731 words.

Table S17. Inter-item correlations for the county-level and individual-level outcomes and controls.

(a) Inter-item correlations for the well-being and health measurements at the county-level.

N = 1208 counties	Life Satisfaction	Happiness	Worry	Sadness	%Population under 18 yrs	% Population over 65 yrs	Median age	%Population African American	Socioeconomic index	% Fair/poor health	All cause mortality	Mentally unhealthy days
Life Satisfaction	1.00	.55	-.41	-.55	.05	-.20	-.24	.08	.59	-.42	-.51	-.44
Happiness	.55	1.00	-.51	-.62	.09	-.09	-.12	-.13	.35	-.45	-.40	-.39
Worry	-.41	-.51	1.00	.68	.00	-.06	-.04	-.05	-.21	.38	.29	.38
Sadness	-.55	-.62	.68	1.00	-.05	.17	.15	.06	-.50	.61	.52	.49
% Population under 18 yrs	.05	.09	.00	-.05	1.00	-.59	-.49	.11	-.09	.12	.10	-.09
% Population over 65 yrs	-.20	-.09	-.06	.17	-.59	1.00	.89	-.24	-.22	.10	.07	.19
Median age	-.24	-.12	-.04	.15	-.49	.89	1.00	-.25	-.06	.01	.02	.15
% Population African	.08	-.13	-.05	.06	.11	-.24	-.25	1.00	-.06	.20	.30	.01
Socioeconomic index	.59	.35	-.21	-.50	-.09	-.22	-.06	-.06	1.00	-.65	-.70	-.44
% Fair/poor health	-.42	-.45	.38	.61	.12	.10	.01	.20	-.65	1.00	.64	.59
All cause mortality	-.51	-.40	.29	.52	.10	.07	.02	.30	-.70	.64	1.00	.49
Mentally unhealthy days	-.44	-.39	.38	.49	-.09	.19	.15	.01	-.44	.59	.49	1.00

(b) Inter-item correlations for the well-being measurements and individual-level.

N = 2321 Facebook users	Life Satisfaction	Happiness	Worry	Sadness
Life Satisfaction	1.00	.66	-.42	-.46
Happiness	.66	1.00	-.47	-.58
Worry	-.42	-.47	1.00	.67
Sadness	-.46	-.58	.67	1.00

Table S18. Inter-item correlations among the Gallup well-being outcomes for 2012-2013 and 2015-2016, for n = 373 counties.

		2012 - 2013				2015 - 2016			
	N = 1208 counties	Life Satisfaction	Happiness	Worry	Sadness	Life Satisfaction	Happiness	Worry	Sadness
Gallup outcomes 2012 - 2013	Life Satisfaction	1.00	.43	-.39	-.48	.65	.36	-.18	-.30
	Happiness	.43	1.00	-.45	-.53	.46	.51	-.31	-.36
	Worry	-.39	-.45	1.00	.62	-.33	-.29	.50	.32
	Sadness	-.48	-.53	.62	1.00	-.46	-.41	.40	.46
Gallup outcomes 2015 - 2016	Life Satisfaction	.65	.46	-.33	-.46	1.00	.59	-.40	-.50
	Happiness	.36	.51	-.29	-.41	.59	1.00	-.35	-.52
	Worry	-.18	-.31	.50	.40	-.40	-.35	1.00	.65
	Sadness	-.30	-.36	.32	.46	-.50	-.52	.65	1.00

Table S19. Inter-item correlations for other LIWC dictionaries which contain positive emotion words.

		Informal language			Personal concerns						
	**LIWC 2015 dictionaries**	**Swear words**	**Assent**	**Netspeak**	**Religion**	**Leisure**	**Work**	**Money**	**Social processes**	**Perceptual processes**	**Biological processes**
Informal language	**Swear words**	1.00	.18	.83	.21	-.55	-.49	-.30	.29	.13	.70
	**Assent**	.18	1.00	.50	.15	-.65	-.67	-.75	.79	-.36	.16
	**Netspeak**	.83	.50	1.00	.38	-.75	-.67	-.52	.46	-.14	.43
Personal concerns	**Religion**	.21	.15	.38	1.00	-.39	-.28	-.23	.25	-.08	-.08
	**Leisure**	-.55	-.65	-.75	-.39	1.00	.76	.66	-.69	.21	-.38
	**Work**	-.49	-.67	-.67	-.28	.76	1.00	.76	-.75	-.01	-.48
	**Money**	-.30	-.75	-.52	-.23	.66	.76	1.00	-.72	.19	-.34
	**Social processes**	.29	.79	.46	.25	-.69	-.75	-.72	1.00	.03	.48
	**Perceptual processes**	.13	-.36	-.14	-.08	.21	-.01	.19	.03	1.00	.45
	**Biological processes**	.70	.16	.43	-.08	-.38	-.48	-.34	.48	.45	1.00

Table S20. Inter-correlations between Twitter's emotion and well-being measurements, calculated at the county-level.

			Word-level approaches													Sentence-level approaches											Person-level models
			LIWC 2015						PERMA lexicon		ANEW		Warr.	LabMT		NRC Hashtag Emotion								Swiss Cheese		WWBP Lexicon	Life Sat.
	**Lexicon**	**Category**	Pos	Pos^	Neg	Sad	Ang	Anx	P+	P-	Val	Val^	Val	Val	Val^	Ant	Joy	Surp	Trust	Fear	Sad	Ang	Disg	Pos	Neg	Affect	
**Word-level approaches**	**LIWC 2015**	(Pos) Positive Emotion	1.00	.66	.26	.31	.16	.17	-.02	.29	.42	.09	-.14	.51	.08	-.45	-.10	-.23	.28	-.42	.19	.22	.13	.05	-.17	.44	-.33
		(Pos^) Positive Emotion modified	.66	1.00	-.28	.28	-.42	.14	.59	.07	.47	.37	.16	.33	.45	.02	.41	.19	-.28	.12	-.38	-.32	-.37	.50	.44	-.26	.02
		(Neg) Negative Emotion	.26	-.28	1.00	.49	.94	.45	-.79	.73	-.23	-.49	-.19	.47	-.20	-.78	-.78	-.51	.46	-.70	.59	.97	.87	-.90	-.91	.83	-.90
		(Sad) Sadness	.31	.28	.49	1.00	.20	.66	-.20	.72	.07	-.22	.00	.59	.20	-.60	-.19	-.46	.24	-.39	.23	.40	.30	-.46	-.32	.25	-.68
		(Ang) Anger	.16	-.42	.94	.20	1.00	.21	-.82	.53	-.29	-.47	-.21	.31	-.29	-.64	-.80	-.43	.44	-.65	.60	.94	.89	-.84	-.87	.82	-.76
		(Anx) Anxiety	.17	.14	.45	.66	.21	1.00	-.15	.76	.02	-.16	.07	.50	.20	-.51	-.26	-.16	-.01	-.19	-.03	.40	.27	-.47	-.43	.24	-.58
	**PERMA lexicon**	(Pos) Positive Emotion	-.02	.59	-.79	-.20	-.82	-.15	1.00	-.41	.43	.63	.49	-.03	.59	.66	.83	.58	-.56	.71	-.70	-.78	-.71	.81	.80	-.78	.62
		(Neg) Negative Emotion	.29	.07	.73	.72	.53	.76	-.41	1.00	-.16	-.42	-.14	.49	-.01	-.73	-.56	-.33	.10	-.48	.19	.69	.49	-.68	-.67	.53	-.82
	**ANEW**	(Val) Valence	.42	.47	-.23	.07	-.29	.02	.43	-.16	1.00	.87	.43	.52	.54	.05	.53	.09	.12	.15	-.13	-.23	-.13	.41	.23	-.15	.15
		(Val^) Valence modified	.09	.37	-.49	-.22	-.47	-.16	.63	-.42	.87	1.00	.62	.29	.65	.43	.70	.42	-.17	.49	-.38	-.47	-.27	.58	.46	-.46	.42
	**(Warr) Warriner's lexicon**	(Val) Valence	-.14	.16	-.19	.00	-.21	.07	.49	-.14	.43	.62	1.00	.50	.91	.51	.52	.50	-.23	.67	-.31	-.14	.04	.19	.23	-.45	.12
	**LabMT**	(Val) Valence	.51	.33	.47	.59	.31	.50	-.03	.49	.52	.29	.50	1.00	.64	-.39	-.01	-.20	.26	-.16	.14	.47	.47	-.31	-.37	.32	-.57
	**LabMT**	(Val^) Valence modified	.08	.45	-.20	.20	-.29	.20	.59	-.01	.54	.65	.91	.64	1.00	.38	.59	.43	-.30	.57	-.40	-.18	-.06	.26	.29	-.43	.03
**Sentence-level approaches**	**NRC Hashtag Emotion**	(Ant) Anticipation	-.45	.02	-.78	-.60	-.64	-.51	.66	-.73	.05	.43	.51	-.39	.38	1.00	.69	.68	-.44	.86	-.46	-.74	-.54	.68	.74	-.81	.79
		(Joy) Joy	-.10	.41	-.78	-.19	-.80	-.26	.83	-.56	.53	.70	.52	-.01	.59	.69	1.00	.40	-.32	.71	-.44	-.79	-.66	.79	.79	-.77	.67
		(Surp) Surprise	-.23	.19	-.51	-.46	-.43	-.16	.58	-.33	.09	.42	.50	-.20	.43	.68	.40	1.00	-.72	.75	-.60	-.49	-.34	.49	.40	-.58	.44
		Trust	.28	-.28	.46	.24	.44	-.01	-.56	.10	.12	-.17	-.23	.26	-.30	-.44	-.32	-.72	1.00	-.61	.77	.45	.50	-.39	-.38	.52	-.29
		Fear	-.42	.12	-.70	-.39	-.65	-.19	.71	-.48	.15	.49	.67	-.16	.57	.86	.71	.75	-.61	1.00	-.60	-.65	-.55	.59	.59	-.81	.65
		(Sad) Sadness	.19	-.38	.59	.23	.60	-.03	-.70	.19	-.13	-.38	-.31	.14	-.40	-.46	-.44	-.60	.77	-.60	1.00	.56	.56	-.49	-.55	.62	-.39
		(Ang) Anger	.22	-.32	.97	.40	.94	.40	-.78	.69	-.23	-.47	-.14	.47	-.18	-.74	-.79	-.49	.45	-.65	.56	1.00	.87	-.89	-.91	.82	-.86
		(Disg) Disgust	.13	-.37	.87	.30	.89	.27	-.71	.49	-.13	-.27	.04	.47	-.06	-.54	-.66	-.34	.50	-.55	.56	.87	1.00	-.81	-.77	.68	-.76
	**Swiss Chocolate**	(Pos) Positive Emotion	.05	.50	-.90	-.46	-.84	-.47	.81	-.68	.41	.58	.19	-.31	.26	.68	.79	.49	-.39	.59	-.49	-.89	-.81	1.00	.86	-.71	.81
		(Neg) Negative Emotion	-.17	.44	-.91	-.32	-.87	-.43	.80	-.67	.23	.46	.23	-.37	.29	.74	.79	.40	-.38	.59	-.55	-.91	-.77	.86	1.00	-.84	.75
	**WWBP lexicon**	Affect	.44	-.26	.83	.25	.82	.24	-.78	.53	-.15	-.46	-.45	.32	-.43	-.81	-.77	-.58	.52	-.81	.62	.82	.68	-.71	-.84	1.00	-.70
**Person-level models**	**WWBP Life Satisfaction**		-.33	.02	-.90	-.68	-.76	-.58	.62	-.82	.15	.42	.12	-.57	.03	.79	.67	.44	-.29	.65	-.39	-.86	-.76	.81	.75	-.70	1.00

References

1. Sharecare (2020) Gallup-sharecare well-being index.
2. Pennebaker JW, Boyd RL, Jordan K, Blackburn K (2015) The development and psychometric properties of liwc2015, Technical report.
3. Seligman ME (2012) *Flourish: A visionary new understanding of happiness and well-being.* (Simon and Schuster).
4. Schwartz HA, et al. (2013) Choosing the right words: Characterizing and reducing error of the word count approach in *Second Joint Conference on Lexical and Computational Semantics (* SEM), Volume 1: Proceedings of the Main Conference and the Shared Task: Semantic Textual Similarity.* Vol. 1, pp. 296–305.
5. Bradley MM, Lang PJ (1999) Affective norms for english words (anew): Instruction manual and affective ratings, (Citeseer), Technical report.
6. Warriner AB, Kuperman V, Brysbaert M (2013) Norms of valence, arousal, and dominance for 13,915 english lemmas. *Behavior research methods* 45(4):1191–1207.
7. Dodds PS, Harris KD, Kloumann IM, Bliss CA, Danforth CM (2011) Temporal patterns of happiness and information in a global social network: Hedonometrics and twitter. *PloS one* 6(12):e26752.
8. Mohammad SM, Kiritchenko S (2015) Using hashtags to capture fine emotion categories from tweets. *Computational Intelligence* 31(2):301–326.
9. Jaggi M, Uzdilli F, Cieliebak M (2014) Swiss-chocolate: Sentiment detection using sparse svms and part-of-speech n-grams in *Proceedings of the 8th International Workshop on Semantic Evaluation (SemEval 2014).* pp. 601–604.
10. Rieman D, Jaidka K, Schwartz HA, Ungar L (2017) Domain adaptation from user-level facebook models to county-level twitter predictions in *Proceedings of the Eighth International Joint Conference on Natural Language Processing (Volume 1: Long Papers).* pp. 764–773.
11. Guntuku SC, Buffone A, Jaidka K, Eichstaedt JC, Ungar LH (2019) Understanding and measuring psychological stress using social media in *Proceedings of the International AAAI Conference on Web and Social Media.* Vol. 13, pp. 214–225.
12. Preoţiuc-Pietro D, et al. (2016) Modelling valence and arousal in facebook posts in *Proceedings of the 7th Workshop on Computational Approaches to Subjectivity, Sentiment and Social Media Analysis.* pp. 9–15.
13. Schwartz HA, et al. (2013) Personality, gender, and age in the language of social media: The open-vocabulary approach. *PloS one* 8(9):e73791.
14. Park G, et al. (2015) Automatic personality assessment through social media language. *Journal of personality and social psychology* 108(6):934.
15. Blei D, Ng A, Jordan M (2003) Latent dirichlet allocation journal of machine learning research. 3:993–1022.
16. Jaidka K, Guntuku SC, Buffone A, Schwartz HA, Ungar L (2018) Facebook vs. twitter: Differences in self-disclosure and trait prediction in *Proceedings of the International AAAI Conference on Web and Social Media.*
17. Jaidka K, Zhou A, Lelkes Y (2019) Brevity is the soul of twitter: The constraint affordance and political discussion. *Journal of Communication* 69(4):345–372.
18. Guntuku SC, Yaden DB, Kern ML, Ungar LH, Eichstaedt JC (2017) Detecting depression and mental illness on social media: an integrative review. *Current Opinion in Behavioral Sciences* 18:43–49.
19. Sap M, et al. (2014) Developing age and gender predictive lexica over social media in *Proceedings of the 2014 Conference on Empirical Methods in Natural Language Processing (EMNLP).* (Association for Computational Linguistics, Doha, Qatar), pp. 1146–1151.
20. Bureau UC (2015) 2010-2015 american community survey 5-year estimates.
21. Howell RT, Howell CJ (2008) The relation of economic status to subjective well-being in developing countries: A meta-analysis. *Psychological bulletin* 134(4):536.
22. Jebb AT, Tay L, Diener E, Oishi S (2018) Happy states of america: A state level analysis of psychological, economic and social well-being. *Nature Human Behaviour* 2(1):33.
23. Kahneman D, Deaton A (2010) High income improves evaluation of life but not emotional well-being. *Proceedings of the national academy of sciences* 107(38):16489–16493.
24. Eichstaedt JC, et al. (2015) Psychological language on twitter predicts county-level heart disease mortality. *Psychological science* 26(2):159–169.
25. Hoover J, Dehghani M (2018) The big, the bad, and the ugly: Geographic estimation with flawed psychological data. *PsyArXiv.*
26. Giorgi S, Ungar LH, Schwartz HA (In press) Correcting sociodemographic selection biases for population prediction. *arXiv preprint.*
27. Holt D, Smith TF (1979) Post stratification. *Journal of the Royal Statistical Society: Series A (General)* 142(1):33–46.
28. Little RJ (1993) Post-stratification: a modeler's perspective. *Journal of the American Statistical Association* 88(423):1001–1012.
29. Gelman A, Little TC (1997) Poststratification into many categories using hierarchical logistic regression.
30. Henry K, Valliant R (2012) Methods for adjusting survey weights when estimating a total. *Proceedings of the Federal Committee on Statistical Methodology, January* pp. 10–12.
31. Deville JC, Särndal CE, Sautory O (1993) Generalized raking procedures in survey sampling. *Journal of the American statistical Association* 88(423):1013–1020.
32. Fienberg SE, , et al. (1970) An iterative procedure for estimation in contingency tables. *The Annals of Mathematical*

Statistics 41(3):907–917.
33. Kalton G, Flores-Cervantes I (2003) Weighting methods. *Journal of official statistics* 19(2):81.
34. Stephan FF (1942) An iterative method of adjusting sample frequency tables when expected marginal totals are known. *Ann. Math. Statist.* 13(2):166–178.
35. Bureau UC (2010) Census regions and divisions of the united states. *US Census Bureau website.*
36. Giorgi S, et al. (2018) The remarkable benefit of user-level aggregation for lexical-based population-level predictions in *Proceedings of the 2018 Conference on Empirical Methods in Natural Language Processing.*
37. Matz SC, Menges JI, Stillwell DJ, Schwartz HA (2019) Predicting individual-level income from facebook profiles. *PloS one.*
38. Jaidka K, Eichstaedt jC, Giorgi S (2020) Data and resources for estimating geographic subjective well-being from twitter: a comparison of dictionary and data-driven language methods.
39. Rahman J (2012) The n word: Its history and use in the african american community. *Journal of English Linguistics* 40(2):137–171.
40. McCulloch G (2019) *Because Internet: Understanding the New Rules of Language.* (Riverhead Books).
41. Florini S (2014) Tweets, tweeps, and signifyin' communication and cultural performance on "black twitter". *Television & New Media* 15(3):223–237.
42. Brock A (2012) From the blackhand side: Twitter as a cultural conversation. *Journal of Broadcasting & Electronic Media* 56(4):529–549.
43. Pennebaker JW, Lay TC (2002) Language use and personality during crises: Analyses of mayor rudolph giuliani's press conferences. *Journal of Research in Personality* 36(3):271–282.
44. Hitlin P (2016) Turkers in this canvassing: young, well-educated and frequent users. *Pew Research Center.*
